# Diastereotopic groups in two new single-enanti­omer structures (*R*_2_)P(O)[NH-(+)CH(C_2_H_5_)(C_6_H_5_)] (*R* = OC_6_H_5_ and C_6_H_5_)

**DOI:** 10.1107/S2056989023006278

**Published:** 2023-08-01

**Authors:** Farnaz Eslami, Mehrdad Pourayoubi, Fahimeh Sabbaghi, Eliška Skořepová, Michal Dušek, Sahar Baniyaghoob

**Affiliations:** a Department of Chemistry, Science and Research Branch, Islamic Azad University, Tehran, Iran; b Department of Chemistry, Faculty of Science, Ferdowsi University of Mashhad, Mashhad, Iran; c Department of Chemistry, Zanjan Branch, Islamic Azad University, Zanjan, Iran; d Institute of Physics of the Czech Academy of Sciences, Na Slovance 2, 182 21, Prague 8, Czech Republic; Universidad Nacional Autónoma de México, México

**Keywords:** phospho­ramide, phosphinamide, single-enanti­omer, diastereotopic groups, X-ray crystallography, crystal structure

## Abstract

The crystal structures of two single-enanti­omer compounds, *i.e.* diphenyl [(*R*)-(+)-α-ethyl­benzyl­amido]­phosphate, C_21_H_22_NO_3_P or (C_6_H_5_O)_2_P(O)[NH-(*R*)-(+)CH(C_2_H_5_)(C_6_H_5_)] (**I**), and *N*-[(*R*)-(+)-α-ethyl­benz­yl]-*P*,*P*-di­phenyl­phosphinic amide, C_21_H_22_NOP or (C_6_H_5_)_2_P(O)[NH-*R*-(+)CH(C_2_H_5_)(C_6_H_5_)] (**II**), were studied. The different environments at the phospho­rus atoms, (O)_2_P(O)(N) and (C)_2_P(O)(N), allow the P=O/P—N bond strengths to be compared, as well as the N—H⋯O=P hydrogen-bond strengths, and P=O/N—H vibrations. The following characteristics related to diastereotopic C_6_H_5_O/C_6_H_5_ groups in **I**/**II** were considered: geometry parameters, contributions to the crystal packing, solution ^13^C/^1^H NMR chemical shifts, conformations, and NMR coupling constants. The phospho­rus-carbon coupling constants *^n^J*_PC_ (*n* = 2 and 3) in **I** and *^m^J*_PC_ (*m* = 1, 2, 3 and 4) in **II** were evaluated. For a comparative study, chiral analogous structures were retrieved from the Cambridge Structural Database (CSD) and their geometries and conformations are discussed.

## Abstract 2

Two new single-enanti­omer phospho­rus structures were studied. Their geometries, conformations and NMR features are discussed.

## 1. Chemical context

Phospho­ramide/phosphinamide moieties are well-known structural motifs of some bioactive products and drugs (Warren *et al.*, 2016 ▸; Palacios *et al.*, 2005 ▸). There are also reports on their applications in flame retardants (Nguyen & Kim, 2008 ▸), ligands (Wang *et al.*, 2021 ▸; Ferentinos *et al.*, 2019 ▸; Zhang *et al.*, 2019 ▸), extractants (Akbari *et al.*, 2019 ▸), anion transporters (Cranwell *et al.*, 2013 ▸) and catalysts (Klare *et al.*, 2014 ▸). 
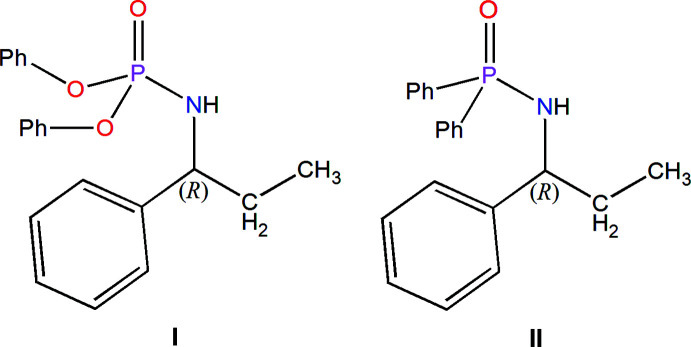


Some of these characteristics are general for phospho­r­amide/phosphinamide compounds, and can be influenced by the groups attached to the common NP=O unit. Typically, the donor property of the phosphoryl group is beneficial in sorption processes, inter­actions with some enzymes and the formation of hydrogen bonds (Corbridge, 2000 ▸). The subfamily to which the compounds belong also plays a role. For example, phosphinicamides with the (C)_2_P(O)(N) skeleton are found to have higher electron-donor properties with respect to amido­phospho­diesters with the (O)_2_P(O)(N) skeleton. The chirality may also be essential for some partic­ular applications, such as the manufacture of drugs and the planning of some reactions related to different reactivities of diastereotopic groups (Nakayama & Thompson, 1990 ▸), enanti­oseparation (Ahmadabad *et al.*, 2019 ▸) and enanti­o­selective catalysis (Liao *et al.*, 2019 ▸).

Recently, we have reported some single-enanti­omer small mol­ecules, belonging to the phospho­ramide family, and phosphor­amide-based macromolecules/hydro­gels (Ahmadabad *et al.*, 2019 ▸; Taherzadeh *et al.*, 2021 ▸; Sabbaghi *et al.*, 2019 ▸). The related synthesis procedure could also be developed for manufacturing phosphinamide-based materials. Moreover, we are inter­ested in studying the differences between two diastereotopic groups in chiral structures. The reason for such attention is the asymmetric induction at phospho­rus by the chiral group, which causes different reactivities of two diastereotopic groups (Nakayama & Thompson, 1990 ▸). These differences were investigated in organic syntheses for the creation of new stereocentres and also can be used for the design and synthesis of ligands with different donor properties of the diastereotopic groups.

In the present work, we continue with the synthesis of new chiral (C_6_H_5_O)_2_P(O)[NH-(+)CH(C_2_H_5_)(C_6_H_5_)] phospho­r­amide, (**I**), and (C_6_H_5_)_2_P(O)[NH-(+)CH(C_2_H_5_)(C_6_H_5_)] phosphinamide, (**II**) to study structural differences of two diastereotopic C_6_H_5_O/C_6_H_5_ groups, caused by the same chiral amine. Structure **I** is the enanti­omer of the previously reported (C_6_H_5_O)_2_P(O)[NH-(–)CH(C_2_H_5_)(C_6_H_5_)] (Sabbaghi *et al.*, 2011 ▸). The investigation is completed by considering structural differences/similarities of diastereotopic groups in analogous chiral structures retrieved from the CSD (Groom *et al.*, 2016 ▸). The main features of the NMR parameters of the diastereotopic groups in **I** and **II** are also discussed.

## 2. Structural commentary

**Figure 1:**
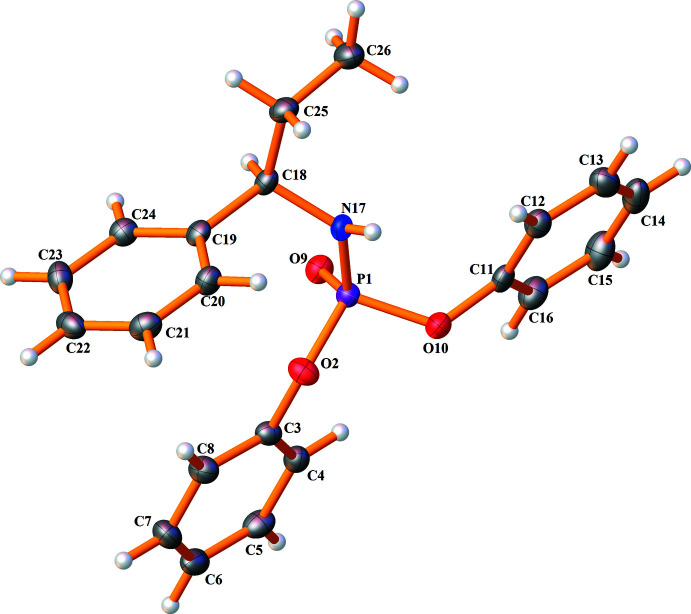
The asymmetric unit of **I**, showing the atom-numbering scheme for non-hydrogen atoms and displacement ellipsoids at 50% probability level. Hydrogen atoms are drawn as spheres of arbitrary radii.

**Figure 2:**
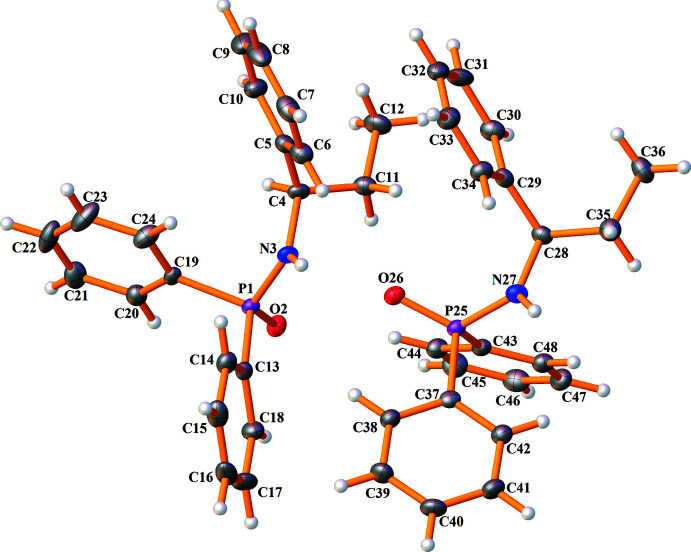
Displacement ellipsoid plot (50% probability) of the asymmetric unit of **II**, showing the atom-numbering scheme for non-hydrogen atoms. Hydrogen atoms are drawn as spheres of arbitrary radii.

Compound **I** crystallizes in the ortho­rhom­bic chiral space group *P*2_1_2_1_2_1_, with the asymmetric unit composed of one amido­phospho­diester mol­ecule (Fig. 1 ▸). Compound **II** is triclinic in chiral space group *P*1, and its asymmetric unit consists of two phosphinicamide mol­ecules (Fig. 2 ▸). Selected bond lengths and angles are presented in Tables 1 ▸ and 2 ▸. All bond distances and angles are within the values observed in analogous structures (Vahdani Alviri *et al.*, 2020 ▸; Hamzehee *et al.*, 2017 ▸).

The P atoms display a distorted tetra­hedral environment, (O)_2_P(O)(N) for **I** and (C)_2_P(O)(N) for **II**, and the maximum/minimum bond angles at phospho­rus are related to O=P—O/O—P—O and O=P—N/N—P—C. The differences between maximum and minimum values are about 16.8° for **I** and 17.5°/16.9° for the two symmetry-independent mol­ecules of **II**. The P—N—C angles in **I** and **II**, for example, P1—N3—C4 angle in **II** of 120.91 (14)° (Table 2 ▸), demonstrate that the hybridization state of nitro­gen atoms is close to *sp* ^2^. The P—O—C angles of **I**, 127.68 (17)°/121.91 (16)°, similarly show the hybridization state of the ester oxygen atoms is close to *sp* ^2^.

**Figure 3:**
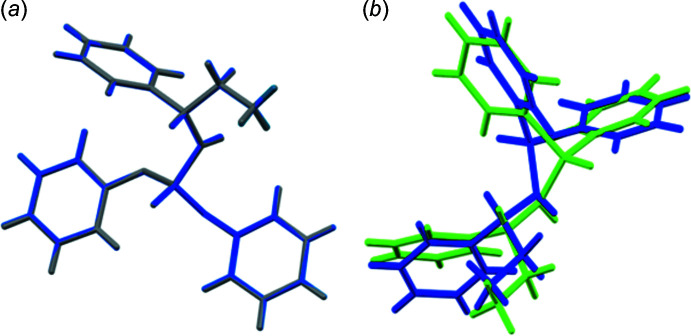
(*a*) Overlay of EXIQIM (grey) and inverted **I** (blue). (*b*) Overlay of two symmetry-independent mol­ecules of **II** (green and blue show mol­ecules P1 and P25, respectively).

The structure **I** is similar to its *S*-enanti­omer (EXIQIM; Sabbaghi *et al.*, 2011 ▸) regarding space group, unit cell and other structural parameters; the only substantial difference is related to the configuration at dissymmetric carbon atoms. Fig. 3 ▸ *a* shows the overlay of the inverted structure of **I** with EXIQIM. The overlay is calculated with a root-mean-square deviation (r.m.s.d.) of 0.0089 Å and a maximum deviation of 0.0153 Å.

The P=O bond in **I**, 1.469 (2) Å, is shorter than the P=O bonds in **II**, 1.4846 (15)/1.4933 (15) Å, and the same is true about the P—N bonds [1.619 (2) Å in **I**, and 1.6367 (19)/1.6426 (19) Å in **II**]. The differences result from the effect of electronegative oxygen atoms of two C_6_H_5_O groups in **I** attached to phospho­rus, while in **II**, there are two C_6_H_5_ groups. The longer P—N bond in **II** is also caused by the steric effects of two phenyl groups directly attached to phospho­rus. Minor differences are observed for the bond lengths related to the diastereotopic pairs. Typically, the P—O and P—C bonds in **I** and two independent mol­ecules of **II** are 1.581 (2)/1.592 (2) Å, 1.802 (2)/1.808 (2) Å and 1.808 (2)/1.797 (2) Å.

In compound **I**, the N—H unit adopts an anti­periplanar (–*ap*) orientation with respect to the P=O group (based on the O=P—N—H torsion angle of −157.18°), and in two symmetry-independent mol­ecules of **II**, the same units adopt synclinal (–*sc* and +*sc*) conformations (the torsion angles are −80.63° and +84.78°). The different conformation of **II** (in comparison to **I**) results from intra­molecular rotations of the chiral amine, and the two independent mol­ecules feature different rotations, but with a similar O=P—N—H conformation.

In **II**, the symmetry-independent mol­ecules are similar concerning the bond lengths and angles (see Table 2 ▸). However, they show some differences in torsion angles (and conformations). Typically, the conformations in the CH_3_—CH_2_—CH—NH—P=O segment are defined by the C—C—C—N/C—C—N—P/C—N—P=O torsion angles, and the values in the P1 molecule of +173.7 (2)°/−98.2 (2)°/60.7 (2)° correspond to +*ap*/−*ac*/+*sc* conformations (*ac* = anti­clinal). Similar torsion angles in the other mol­ecule, −178.3 (2)°/−158.6 (2)°/−62.0 (2)°, define −*ap*/–*ap*/−*sc* conformations. The other notable difference between the two mol­ecules is reflected in the direction of the phenyl ring of the chiral segment with respect to the P=O group (an opposite direction in the mol­ecule P1 and the same direction in the second mol­ecule). Fig. 3 ▸ *b* shows the overlay of two mol­ecules, and the root-mean-square deviation (r.m.s.d.) of the fit of them is 1.3533 Å with a maximum deviation of 4.6684 Å. The noted difference is reflected in the spatial distances of phenyl groups bonded to P and the phenyl group of chiral amine in the two mol­ecules. The differences in diastereotopic phenyl rings in each mol­ecule can also be described by their distances from the phenyl ring of the chiral amine.

For **I**, the distances between the centroid of the phenyl ring of chiral amine and the centroids of two diastereotopic phenyl groups are 5.0848 (1) and 7.9514 (1) Å. For the two symmetry-independent mol­ecules of **II**, equivalent distances are 5.5767 (5)/7.0325 (6) Å and 7.1614 (6)/6.4951 (3) Å. These spatial distances show that one of the diastereotopic phenyl rings is significantly closer to the phenyl of the chiral amine. The differences in these spatial distances are pronounced in **I**, where the flexibility is greater (because of the existence of the P—O—C segment and the possibility of rotation).

In **I**, the conformations of phenyl rings can be introduced by the C—C—O—P torsion angles, which are 32.1 (3)°/−149.9 (2)° and 86.7 (3)°/−98.0 (3)° according to the +*sc*−*ac* conformations for both phenyl rings. In the structure of **II**, the C—C—P=O torsion angles were considered for checking the conformations of the phenyl rings. The values are 173.8 (2)°/−10.4 (2)° and 25.4 (2)°/−158.0 (2)° (+*ap*−*sp* and +*sp*−*ap*) in one mol­ecule and 10.8 (2)°/−169.6 (2)° and −18.4 (2)°/163.0 (2)° (+*sp*−*ap* and −*sp*+*ap*) in the other mol­ecule, which also show similar conformations.

## 3. Supra­molecular features

**Figure 4:**
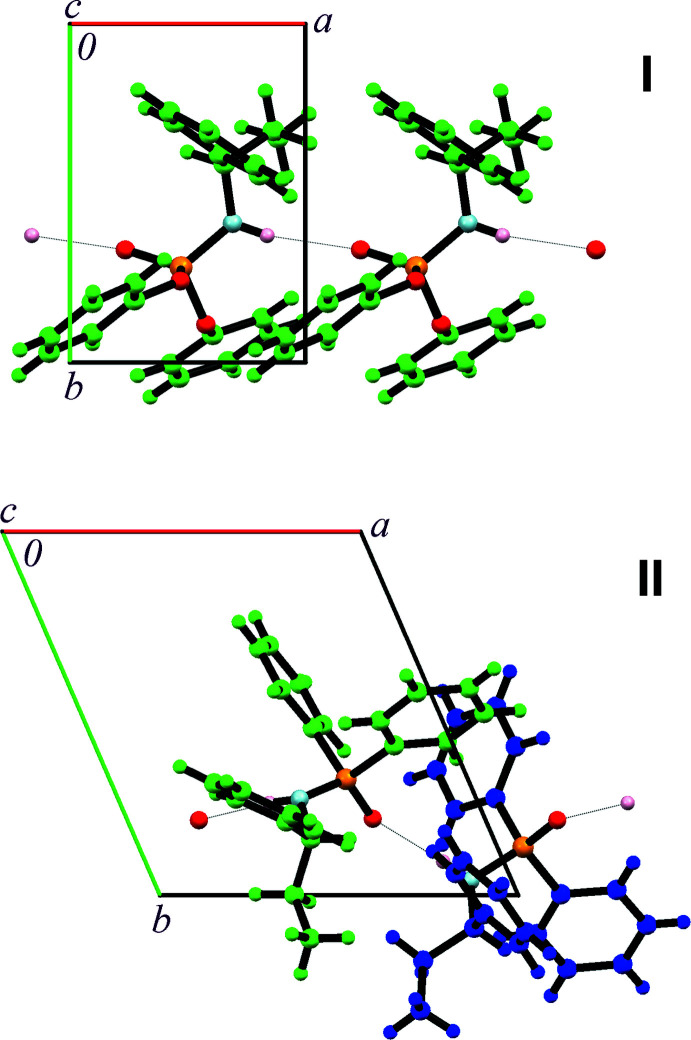
Crystal packing of **I** (top) and **II** (bottom). The red, orange, light-blue and pink balls show oxygen, phospho­rus, nitro­gen and hydrogen attached to nitro­gen atoms. For **I**, carbon atoms and attached hydrogen atoms are shown in light green. For **II**, two-symmetry independent mol­ecules are shown in light green and blue. The dotted lines show N—H⋯O hydrogen bonds.

In the crystal structures of **I** and **II**, the mol­ecules are assembled in a chain arrangement through N—H⋯O(P) hydrogen bonds along [100] (Fig. 4 ▸, Tables 3 ▸ and 4 ▸). The N—H⋯O(P) hydrogen bond in **I** is weaker than in **II** (H⋯O distances are 2.24 and 1.97/2.08 Å, respectively). This weakness is the result of the lower hydrogen-bond acceptor capability expected for the phosphoryl group of an (O)_2_(N)P(O)-based structure, compared to the phosphoryl group of a (C)_2_(N)P(O)-based structure, and is due to the two atoms with a higher electronegativity bonded to the phospho­rus atom. The effect of different electronegativities was previously noted (*see above*) for different P=O bond lengths.

**Figure 5:**
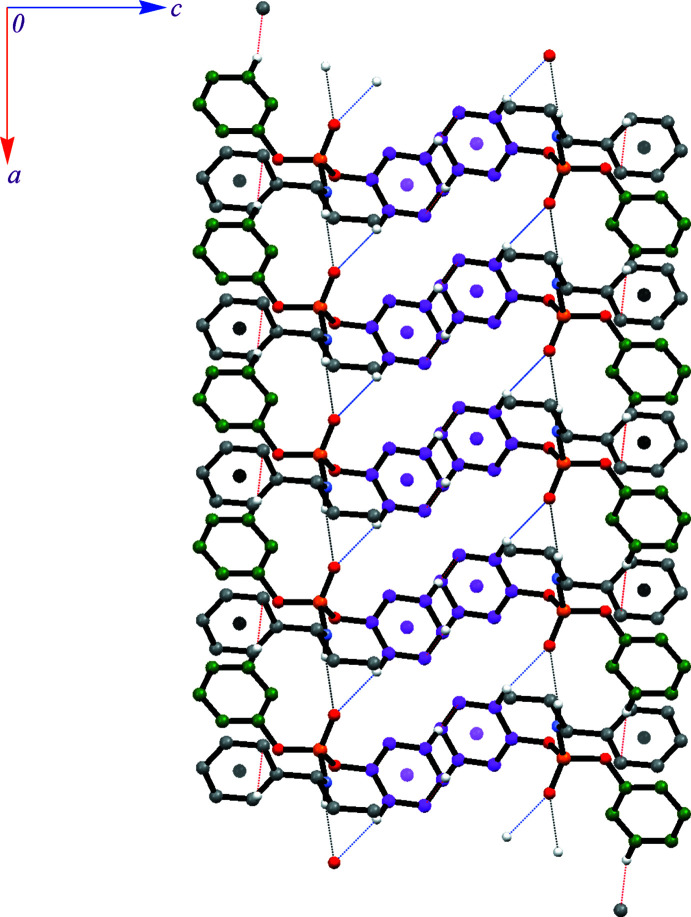
A view of the two-dimensional double-layered arrangement of **I** formed by N—H⋯O, C—H⋯O and C—H⋯π inter­actions (shown as black, blue and red dotted lines, respectively). The centroids of the phenyl rings taking part as acceptors in C—H⋯π inter­actions are shown as balls of the same colours as the corresponding ring.

C—H⋯π inter­actions in **I** assemble the mol­ecules in a two-dimensional array in the *ab* plane. Fig. 5 ▸ shows the mol­ecular assembly formed by the N—H⋯O, C—H⋯O and possible C—H⋯π inter­actions, where the C—H⋯O inter­action does not change the dimensionality made by the N—H⋯O hydrogen bond. To show better the contact(s) contributing by each phenyl ring, the rings are distinguished by colours: green (C3–C8) and magenta (C11–C16) for the diastereotopic rings and grey (C19–C24) for the phenyl ring of the chiral amine. The green ring takes part in a C—H⋯π inter­action as a donor (the acceptor is the grey ring) (H⋯*Cg* = 2.81 Å). The magenta ring takes part in a C—H⋯π inter­action with an adjacent symmetry-related magenta ring (H⋯*Cg* = 3.23 Å) and also in a C—H⋯OP inter­action (H⋯O = 2.55 Å). The formed two-dimensional assembly is double-layered and has a thickness of 18.057 Å in the *c*-axis direction.

**Figure 6:**
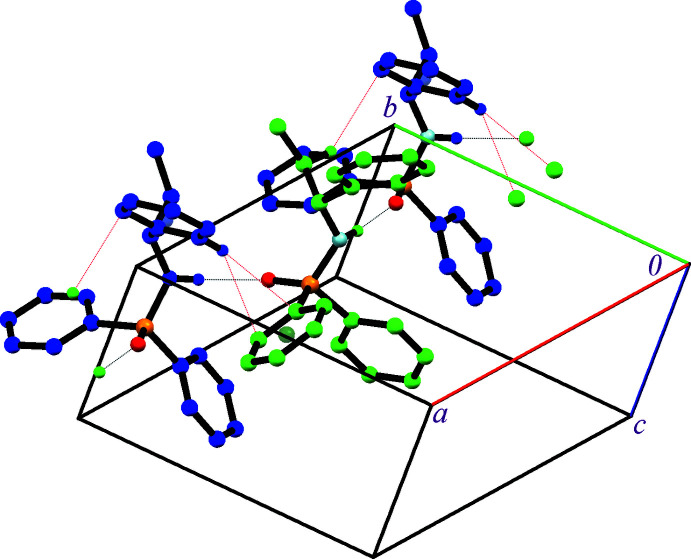
A view of the one-dimensional arrangement of structure **II** formed by N—H⋯O and C—H⋯π inter­actions (shown as black and red dotted lines). Only the hydrogen atoms participating in these hydrogen-bond interactions are shown.

In the structure of **II**, two possible C—H⋯π inter­actions exist (H⋯*Cg* distances of 3.41 and 3.49 Å), which do not change the dimensionality made by the N—H⋯O hydrogen bonds. In both C—H⋯π inter­actions, the H donors are chiral amines of two symmetry-independent mol­ecules (the *ortho*-hydrogen atom and the hydrogen of the CH_2_ unit, as shown in Fig. 6 ▸). The acceptors are one of the diastereotopic phenyl rings of the molecule including atom P1 and the phenyl ring of the chiral amine in the other molecule.

## 4. An overview of diastereotopic groups in analogous structures

The chiral structures with an *R*_2_P(=*X*)—N—C(H)(**C**)(C—C) fragment (*X* = O, S, N; **C** is a dissymmetric carbon atom) were retrieved from the CSD to study possible structural differences for diastereotopic *R* groups; the metal complexes were not considered. The CSD (version 5.42 updated on Feb. 2021; Groom *et al.*, 2016 ▸) comprises 48 such structures, of which two were unavailable. The remaining 46 structures include 79 pairs of diastereotopic P—*Y* (*Y* = C, O, N) bonds, and the structures have different skeletons, (C)_2_P(O)(N), (C)_2_P(S)(N), (C)_2_P(N)(N), (O)_2_P(O)(N) and (N)_2_P(O)(N). The related bond lengths are given in Table S1 of the supporting inform­ation. The largest difference for the P—C bond lengths made by diastereotopic groups (0.025 Å) exceeds the largest differences for the P—O (0.017 Å) and P—N bond lengths (0.015 Å). The P—C, P—N and P—O bond lengths in these structures vary from 1.773 to 1.837 Å, 1.629 to 1.652 Å and 1.555 to 1.607 Å, respectively, with averages of 1.805, 1.643 and 1.580 Å.

The conformations of diastereotopic groups attached to phospho­rus were analysed in the structures analogous to **I** and **II**, *i.e*. with the O_2_P and C_2_P skeletons. Only three O_2_P-based structures (with the oxygen atom attached to an arene ring) were found in the CSD. For the C_2_P skeleton, 36 structures, including 64 *R*_2_P*X* fragments, were checked, and the C—C—P=*X* (*X* = O, N, S) torsion angles were evaluated.

The C_2_P-based structures mainly include Ph_2_P(O) fragment (28 structures), similar to compound **II**; however, structures with Ph_2_P(S) (seven structures), and (C_6_H_11_)_2_P(=N) (one structure) fragments were also found. Both similar and different conformations were observed for diastereotopic groups. Details of the analysis are given in Table S2 and Fig. S1 of the supporting information. The torsion angles such as C—C—P=O of 0.04° in the structure with refcode MEFCIK (Sweeney *et al.*, 2006 ▸) show the P=O group nearly in a plane where the phenyl ring also exists. Its complementary torsion angle for the other C—C—P=O related to this phenyl ring is 176.54°, and these two torsion angles define the *sp*+*ap* conformation of this phenyl ring with respect to the P=O group. On the other hand, most of the structures also include ±*sp*±*ap* conformations at least for one phenyl ring. The most populated conformations for diastereotopic fragments (sep­arated by "/") are ±*sp*±*ap*/±*sp*±*ap* (26 entries) and ±*sp*±*ap*/±*sc*±*ac* (23 entries). In the systems with phenyl rings directly attached to the phospho­rus atom, as a result of crowding, the simultaneous torsion angles around ±90° (a perpendicular conformation) for both phenyl rings were not found for any structure. In some cases, like in the structure with refcode VUGSOG (Yin *et al.*, 2009 ▸) with close phenyl rings, the CH unit of one phenyl ring is directed toward the centroid of the second phenyl ring because of the formation of an intra­molecular C—H⋯π inter­action.

As a result of the existence of C—O—P moiety in the O_2_P-based structures, the flexibility is expected to be higher than for Ph_2_P-based structures; the three structures show different conformations but they include ±*sc*±*ac* conformations at least in one arene ring.

## 5. Hirshfeld surface analyses and fingerprint plots of structures I and II

**Figure 7:**
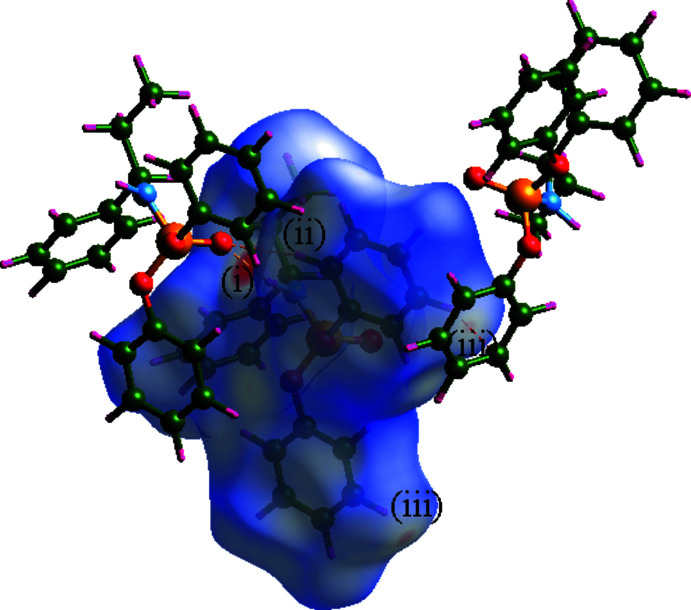
Hirshfeld surface map generated for the structure **I**. Two mol­ecules are shown outside the surface to represent the N—H⋯O [label (i)], C—H⋯O [label (ii)] and typical C—H⋯π [label (iii)] inter­actions with the mol­ecule within the surface.

To visualize and compare the inter­molecular contacts of **I** and **II**, the Hirshfeld surfaces (HS) mapped with *d*_norm_ and two-dimensional fingerprint plots (Spackman & Jayatilaka, 2009 ▸; Spackman *et al.*, 2021 ▸) were generated using the *CrystalExplorer* program (Wolff *et al.*, 2013 ▸). In the HS map of **I** (Fig. 7 ▸), the red areas are associated with the N—H⋯O, C—H⋯O and 2×C—H⋯π inter­actions [labels (i), (ii) and (iii)]. The contacts of **I**, obtained from the fingerprint plots, are H⋯H (57.3%), H⋯C (28.8%), H⋯O (12.7%) and O⋯C (1.2%). The O⋯C contact results from the near distance of two symmetry-related phen­oxy groups [O2(C3–C8)], through the ester oxygen atom and π-system.

**Figure 8:**
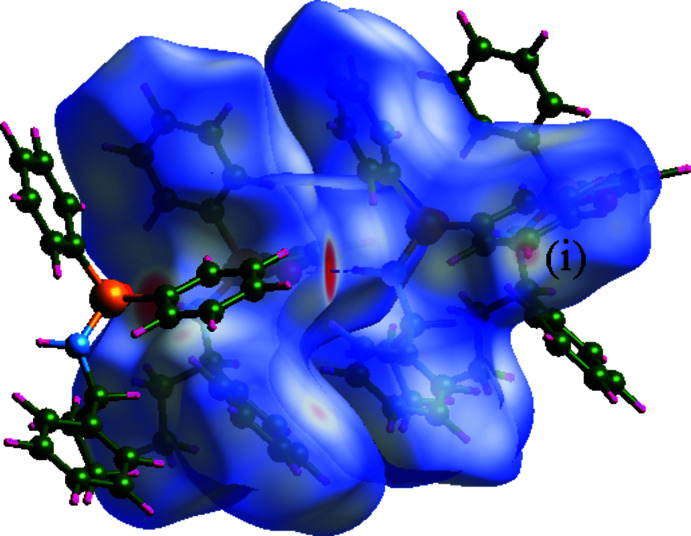
Hirshfeld surface map generated step by step around two symmetry-independent mol­ecules of **II**. The two mol­ecules outside the surface were given to show the hydrogen-bond inter­actions with the mol­ecules within the surface. The red region labelled (i) is related to a close H⋯H contact between the mol­ecules within and outside the surface (not shown).

For **II**, the HS map was generated around two symmetry-independent mol­ecules step by step. Besides N—H⋯O hydrogen bonds, a significant H⋯H contact develops a red area, as seen in Fig. 8 ▸. This inter­action is between H231 of the phenyl ring of mol­ecule P1 connected to H301 of the chiral amine of the other mol­ecule. The H⋯H separation was obtained as 2.291 Å and 2.026 Å in the X-ray and Hirshfeld analyses, respectively (the neutron-normalized CH distance is 1.083 Å in Hirshfeld in comparison with 0.941/0.943 Å in X-ray).

The contribution percentages of various contacts were obtained for the two symmetry-independent mol­ecules. Compared with **I**, the structure of **II** shows fewer H⋯O, H⋯C and O⋯C contacts (7.1%/7.0%, 26.1%/25.6%, 0.1% for both), which were compensated with remarkable H⋯H (64.2%/64.8%), and C⋯C contacts (2.5% for both). The smaller volume/*Z* ratio in **II** is reflected by the crowding, manifested in increased H⋯H contacts and the observation of C⋯C contacts.

## 6. Spectroscopy of I and II

In the IR spectra, the N—H stretching bands are centred at 3268 cm^−1^ for **I** and 3152 cm^−1^ for **II**. The lower NH stretching wave number of **II** is attributed to stronger N—H⋯OP hydrogen bonds as discussed in the X-ray crystallography section. The bands at 1244 cm^−1^ for **I** and 1192 cm^−1^ for **II** are assigned to the P=O vibrations, and the higher wave number for **I** is in accordance with the presence of more electronegative atoms in the (O)_2_P(O)N skeleton [*versus* (C)_2_P(O)N for **II**].

In the ^13^C NMR spectra, the doublet signals at 31.80 p.p.m. (^3^ *J* = 8.1 Hz) for **I** and at 32.62 p.p.m. (^3^ *J* = 4.7 Hz) for **II** correspond to the CH_2_ group. The dissymmetric carbon atom does not show coupling with phospho­rus, and the *ipso*-C atom attached to it, *i.e*. with a three-bond separation from phospho­rus, shows a doublet at 143.04 p.p.m. (^3^ *J* = 3.0 Hz) in **I** and at 145.50 p.p.m. (^3^ *J* = 4.6 Hz) in **II**.

For the two diastereotopic C_6_H_5_O groups in **I**, two sets of carbon signals are observed. For example, the doublets at 150.74/150.92 p.p.m. and 120.12/120.23 p.p.m., with ^2^ *J* = 7.0 Hz for the first pair and ^3^ *J* = 4.0 Hz for the second pair, are associated with the diastereotopic *ipso*-C atoms and diastereotopic *ortho*-C atoms, respectively. All carbon atoms of diastereotopic phenyl groups in compound **II** show couplings with phospho­rus (^1^ *J*, ^2^ *J*, ^3^ *J* and ^4^ *J*).

The doublet signals at 131.74/131.86 p.p.m. (*J* = 1.9/2.1 Hz) are assigned to the *para*-carbon atoms of the phenyl rings with four bonds separation from the phospho­rus atom. The doublets at 132.20/132.38 p.p.m. (*J* = 9.4/9.5 Hz) and at 128.62/128.82 p.p.m. (*J* = 12.2/12.1 Hz) are assigned to the diastereotopic *ortho*- and *meta*-carbon atoms. The doublets centred at 134.44 and 134.77 p.p.m. (*J* = 127.4 and 126.4 Hz) are related to the diastereotopic *ipso*-carbon atoms. The separation of these signals is comparable with previously investigated ^1^ *J* coupling constants for analogous compounds, typically in (C_6_H_5_)_2_P(O)(NH-*cyclo*-C_7_H_13_) with ^1^ *J* = 129.4 Hz (Hamzehee *et al.*, 2017 ▸).

A brief discussion of ^31^P NMR and ^1^H NMR spectroscopy is given in the supporting information (Figures S2 to S11).

## 7. Conclusions

The differences/similarities of diastereotopic pairs, 2×C_6_H_5_O/2×C_6_H_5_, were discussed for two new single-enanti­omer structures, (C_6_H_5_O)_2_P(O)[NH-(+)CH(C_2_H_5_)(C_6_H_5_)] (**I**), and (C_6_H_5_)_2_P(O)[NH-(+)CH(C_2_H_5_)(C_6_H_5_)] (**II**). The pronounced differences are related to the contributions in the crystal packing by diastereotopic groups, especially in the C—H⋯π inter­actions, and the NMR chemical shifts of corresponding ^13^C signals. The geometry parameters, conformations and NMR coupling constants of diastereotopic groups show minor differences (and/or similarities in some cases). In **I** with the O_2_P(O)N skeleton, the shorter P=O/P—N bonds and weaker N—H⋯O=P hydrogen bond are observed with respect to the structure **II** with the C_2_P(O)N skeleton. These structural features, resulting from different electronegativities of atoms, are reflected in the higher stretching frequencies of P=O and N—H bonds in the structure **I** (the latter because of a weaker N—H⋯O=P hydrogen bond). The lower volume/*Z* ratio of **II** is reflected by the crowding and observation of C⋯C contacts and raising H⋯H contacts, while **I** includes more H⋯O and O⋯C contacts. The study of analogous chiral structures retrieved from the CSD shows minor differences in bond lengths for diastereotopic P—C, P—O, and P—N bonds and more significant differences in torsion angles of diastereotopic groups.

## 8. Synthesis and crystallization

**Preparation of (C_6_H_5_O)_2_P(O)[NH-(** ***R*** **)-(+)CH(C_2_H_5_)(C_6_H_5_)], (I).** To a solution of (C_6_H_5_O)_2_P(O)Cl in dry chloro­form, a solution of *R*-(+)-1-phenyl­propyl­amine and tri­ethyl­amine (1:1:1 molar ratio) in the same solvent was added at 273 K. After stirring for 4 h, the solvent was removed in a vacuum, and the obtained solid was washed with distilled water to remove (C_2_H_5_)_3_NHCl. Colourless crystals were obtained from a solution of the title compound in CHCl_3_/CH_3_CN (1:2 *v*/*v*) after slow evaporation at room temperature.

Analytical data: IR (KBr, ν, cm^−1^): 3268, 3063, 3029, 2970, 2929, 2854, 1592, 1492, 1453, 1420, 1244, 1200, 1167, 1058, 1020, 949, 900, 750, 689, 634, 579, 552, 520, 496, 457. ^1^H NMR (400.22 MHz, CDCl_3_): *δ* = 0.84 (*t*, *J* = 7.2 Hz, 3H), 1.81 (*m*, 2H), 3.84 (*t*, *J* = 10.8 Hz, 1H, NH), 4.32 (*m*, 1H), 6.99 (*d*, *J* = 8.4 Hz, 2H), 7.10 (*t*, *J* = 7.2 Hz, 1H), 7.16 – 7.35 (*m*, 12H); ^13^C{^1^H} NMR (100.64 MHz, CDCl_3_): *δ* = 10.59, 31.80 (*d*, *J* = 8.1 Hz), 58.20, 120.12 (*d*, *J* = 4.0 Hz), 120.23 (*d*, *J* = 4.0 Hz), 124.63, 124.83, 126.50, 127.21, 128.44, 129.43, 129.64, 143.04 (*d*, *J* = 3.0 Hz), 150.74 (*d*, *J* = 7.0 Hz), 150.92 (*d*, *J* = 7.0 Hz); ^31^P{^1^H} NMR (162.01 MHz, CDCl_3_): *δ* = −2.16.

**Preparation of (C_6_H_5_)_2_P(O)[NH-(** ***R*** **)-(+)CH(C_2_H_5_)(C_6_H_5_)], (II).** To a solution of (C_6_H_5_)_2_P(O)Cl in dry chloro­form, a solution of *R*-(+)-1-phenyl­propyl­amine and tri­ethyl­amine (1:1:1 mole ratio) in the same solvent was added at 273 K. After stirring for 4 h, the solvent was removed in a vacuum, and the obtained solid was washed with distilled water to remove (C_2_H_5_)_3_NHCl. Colourless crystals were obtained from a solution of the title compound in CHCl_3_/CH_3_CN (1:2 *v*/*v*) after slow evaporation at room temperature.

Analytical data: IR (KBr, ν, cm^−1^): 3152, 3057, 3027, 2962, 2928, 2868, 1488, 1440, 1383, 1337, 1305, 1192, 1117, 1056, 1017, 929, 904, 838, 751, 721, 695, 603, 564, 533. ^1^H NMR (400.22 MHz, DMSO-*d*_6_): *δ* = 0.79 (*t*, *J* = 7.6 Hz, 3H), 1.69 (*m*, 1H), 1.82 (*m*, 1H), 3.84 (*m*, 1H), 5.91 (*t*, *J* = 10.2 Hz, 1H, NH), 7.26 (*m*, 5H), 7.37 (*m*, 2H), 7.50 (*m*, 4H), 7.64 (*m*, 2H), 7.79 (*m*, 2H); ^13^C{^1^H} NMR (100.64 MHz, DMSO-*d*_6_): *δ* = 11.60, 32.62 (*d*, *J* = 4.7 Hz), 57.10, 126.87, 126.97, 128.45, 128.62 (*d*, *J* = 12.2 Hz), 128.82 (*d*, *J* = 12.1 Hz), 131.74 (*d*, *J* = 1.9 Hz), 131.86 (*d*, *J* = 2.1 Hz), 132.20 (*d*, *J* = 9.4 Hz), 132.38 (*d*, *J* = 9.5 Hz), 134.44 (*d*, *J* = 127.4 Hz), 134.77 (*d*, *J* = 126.4 Hz), 145.50 (*d*, *J* = 4.6 Hz); ^31^P{^1^H} NMR (162.01 MHz, DMSO-*d*_6_): *δ* = 21.13.

## 9. Refinement

Crystal data, data collection and structure refinement details are summarized in Table 5 ▸. The H atoms were all located in difference-Fourier maps, but those attached to C atoms were repositioned geometrically. The H atoms were initially refined with soft restraints on the bond lengths and angles to regularize their geometries (C—H in the range 0.93–0.98 Å, N—H in the range 0.86–0.89 Å) and *U*_iso_(H) values in the range 1.2–1.5×*U*_eq_ of the parent atom, after which the positions were refined with riding constraints (Cooper *et al.*, 2010 ▸; Watkin & Cooper, 2016 ▸). The absolute configuration was determined from the refinement of the Flack parameter (Parsons *et al.*, 2013 ▸).

## Supplementary Material

Crystal structure: contains datablock(s) global, I, II. DOI: 10.1107/S2056989023006278/jq2028sup1.cif

Figures (related to the CSD analysis and NMR) and tables (CSD) and a brief discussion about NMR. DOI: 10.1107/S2056989023006278/jq2028sup3.docx

CCDC references: 2283048, 2283049

Additional supporting information:  crystallographic information; 3D view; checkCIF report

## Figures and Tables

**Table 1:** Selected geometric parameters (Å, °) for **I** [Chem scheme1]

P1—O2	1.5814 (18)	O2—C3	1.390 (3)
P1—O9	1.469 (2)	O10—C11	1.411 (3)
P1—O10	1.5924 (19)	N17—C18	1.482 (3)
P1—N17	1.619 (2)		
			
O2—P1—O9	113.77 (11)	O2—C3—C8	114.9 (2)
O2—P1—O10	99.62 (10)	P1—O10—C11	121.91 (16)
O9—P1—O10	116.45 (11)	O10—C11—C12	119.5 (2)
O2—P1—N17	106.00 (11)	O10—C11—C16	118.5 (3)
O9—P1—N17	114.90 (12)	P1—N17—C18	120.34 (18)
O10—P1—N17	104.43 (11)	N17—C18—C19	112.8 (2)
P1—O2—C3	127.68 (17)	N17—C18—C25	108.4 (2)
O2—C3—C4	123.5 (2)		

**Table 2:** Selected geometric parameters (Å, °) for **II** [Chem scheme1]

P1—O2	1.4846 (15)	P25—O26	1.4933 (15)
P1—N3	1.6367 (19)	P25—N27	1.6426 (19)
P1—C13	1.802 (2)	P25—C37	1.808 (2)
P1—C19	1.808 (2)	P25—C43	1.797 (2)
N3—C4	1.474 (2)	N27—C28	1.469 (3)
			
O2—P1—N3	119.94 (9)	O26—P25—N27	119.61 (9)
O2—P1—C13	111.80 (9)	O26—P25—C37	109.99 (9)
N3—P1—C13	102.43 (10)	N27—P25—C37	102.69 (10)
O2—P1—C19	110.23 (9)	O26—P25—C43	110.87 (9)
N3—P1—C19	105.15 (10)	N27—P25—C43	104.84 (10)
C13—P1—C19	106.21 (9)	C37—P25—C43	108.07 (10)
P1—N3—C4	120.91 (14)	P25—N27—C28	122.16 (15)
N3—C4—C5	110.46 (17)	N27—C28—C29	114.02 (17)
N3—C4—C11	110.64 (17)	N27—C28—C35	107.53 (17)
P1—C13—C14	121.84 (17)	P25—C37—C38	117.22 (16)
P1—C13—C18	118.28 (17)	P25—C37—C42	123.28 (17)
P1—C19—C20	119.48 (17)	P25—C43—C44	118.93 (17)
P1—C19—C24	121.36 (18)	P25—C43—C48	122.26 (18)

**Table 3:** Hydrogen-bond geometry (Å, °) for **I** [Chem scheme1]

*D*—H⋯*A*	*D*—H	H⋯*A*	*D*⋯*A*	*D*—H⋯*A*
C12—H121⋯O9^i^	0.94	2.55	3.474 (4)	170
N17—H171⋯O9^i^	0.85	2.24	3.074 (4)	166 (2)

Symmetry code: (i) 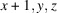 .

**Table 4:** Hydrogen-bond geometry (Å, °) for **II** [Chem scheme1]

*D*—H⋯*A*	*D*—H	H⋯*A*	*D*⋯*A*	*D*—H⋯*A*
N27—H271⋯O2^i^	0.84	2.08	2.923 (4)	176 (2)
N3—H31⋯O26	0.86	1.97	2.817 (4)	173 (2)

Symmetry code: (i) 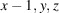 .

**Table 5:** Experimental details

	**I**	**II**
Crystal data		
Chemical formula	C_21_H_22_NO_3_P	C_21_H_22_NOP
*M*_r_	367.38	335.39
Crystal system, space group	Orthorhombic, *P*2_1_2_1_2_1_	Triclinic, *P*1
Temperature (K)	120	95
*a*, *b*, *c* (Å)	5.4947 (1), 8.1503 (1), 41.1096 (7)	9.0483 (7), 10.5533 (8), 11.0036 (6)
α, β, γ (°)	90, 90, 90	70.065 (6), 86.368 (5), 66.571 (7)
*V* (Å^3^)	1841.03 (5)	903.15 (13)
*Z*	4	2
Radiation type	Cu *K*α	Cu *K*α
μ (mm^−1^)	1.49	1.39
Crystal size (mm)	0.90 × 0.27 × 0.07	0.62 × 0.09 × 0.07

Data collection		
Diffractometer	Oxford Diffraction Gemini	Oxford Diffraction SuperNova
Absorption correction	Multi-scan (*CrysAlis PRO*; Rigaku OD, 2017 ▸)	Multi-scan (*CrysAlis PRO*; Rigaku OD, 2017 ▸)
*T*_min_, *T*_max_	0.34, 0.90	0.49, 0.91
No. of measured, independent and observed [*I* > 2.0σ(*I*)] reflections	34246, 3356, 3262	15035, 6781, 6648
*R*_int_	0.067	0.036
(sin θ/λ)_max_ (Å^−1^)	0.626	0.626

Refinement		
*R*[*F* > 2σ(*F*)], *wR*(*F*), *S*	0.037, 0.102, 1.02	0.034, 0.092, 0.97
No. of reflections	3355	6779
No. of parameters	240	443
No. of restraints	4	11
H-atom treatment	H atoms treated by a mixture of independent and constrained refinement	H atoms treated by a mixture of independent and constrained refinement
Δρ_max_, Δρ_min_ (e Å^−3^)	0.33, −0.38	0.49, −0.40
Absolute structure	Parsons *et al.* (2013 ▸), 1324 Friedel pairs	Parsons *et al.* (2013 ▸), 3140 Friedel pairs
Absolute structure parameter	0.013 (9)	−0.013 (7)

Computer programs: *CrysAlis PRO* (Rigaku OD, 2017 ▸), *SUPERFLIP* (Palatinus & Chapuis, 2007 ▸), *CRYSTALS* (Betteridge *et al.*, 2003 ▸), *JANA2006* (Petříček *et al.*, 2014 ▸), *Mercury* (Macrae *et al.*, 2020 ▸) and *MCE* (Rohlíček & Hušák, 2007 ▸).

## supplementary crystallographic information

### Diphenyl [(R)-(+)-α-ethylbenzylamido]phosphate (I) Crystal data

**Table:**

C_21_H_22_NO_3_P	*D*_x_ = 1.325 Mg m^−^^3^
*M**_r_* = 367.38	Cu *K*α radiation, λ = 1.54180 Å
Orthorhombic, *P*2_1_2_1_2_1_	Cell parameters from 19519 reflections
*a* = 5.4947 (1) Å	θ = 4–68°
*b* = 8.1503 (1) Å	µ = 1.49 mm^−^^1^
*c* = 41.1096 (7) Å	*T* = 120 K
*V* = 1841.03 (5) Å^3^	Blade, clear colourless
*Z* = 4	0.90 × 0.27 × 0.07 mm
*F*(000) = 776	

### Diphenyl [(R)-(+)-α-ethylbenzylamido]phosphate (I) Data collection

**Table:**

Oxford Diffraction Gemini diffractometer	3262 reflections with *I* > 2.0σ(*I*)
Graphite monochromator	*R*_int_ = 0.067
ω scans	θ_max_ = 74.9°, θ_min_ = 4.3°
Absorption correction: multi-scan (CrysAlisPro; Rigaku OD, 2017)	*h* = −6→6
*T*_min_ = 0.34, *T*_max_ = 0.90	*k* = −9→9
34246 measured reflections	*l* = −49→48
3356 independent reflections	

### Diphenyl [(R)-(+)-α-ethylbenzylamido]phosphate (I) Refinement

**Table:**

Refinement on *F*^2^	Hydrogen site location: difference Fourier map
Least-squares matrix: full	H atoms treated by a mixture of independent and constrained refinement
*R*[*F* > 3σ(*F*)] = 0.037	Method = Modified Sheldrick *w* = 1/[σ^2^(*F*^2^) + ( 0.03*P*)^2^ + 3.0*P*] , where *P* = (max(*F*_o_^2^,0) + 2*F*_c_^2^)/3
*wR*(*F*) = 0.102	(Δ/σ)_max_ = 0.001
*S* = 1.02	Δρ_max_ = 0.33 e Å^−^^3^
3355 reflections	Δρ_min_ = −0.38 e Å^−^^3^
240 parameters	Absolute structure: Parsons wt al. (2013), 1324 Friedel pairs
4 restraints	Absolute structure parameter: 0.013 (9)
Primary atom site location: other	

### Diphenyl [(R)-(+)-α-ethylbenzylamido]phosphate (I) Special details

**Table:**

Experimental. The crystal was placed in the cold stream of an Oxford Cryosystems open-flow nitrogen cryostat (Cosier & Glazer, 1986) with a nominal stability of 0.1K. Cosier, J. & Glazer, A.M., 1986. J. Appl. Cryst. 105-107.
Refinement. X-ray analyses of I and II were performed on two different diffractometers, both using mirror-collimated Cu-*Kα* radiation (*λ* = 1.5418 Å), and CCD detector Atlas S2. The 120 K data set was acquired on a Gemini diffractometer with a classical sealed X-ray tube, while the 95 K data set was obtained on a SuperNova diffractometer with a micro-focus sealed tube. The data reduction and absorption correction were made with *CrysAlis PRO* software (Rigaku, 2017). The structures were solved by charge flipping methods using *SUPERFLIP* (Palatinus & Chapuis, 2007) software and refined by full-matrix least-squares on *F* squared value using Crystals (Betteridge *et al.*, 2003) and *JANA2006* (Petricek *et al.*, 2014) software programs. MCE (Rohlicek & Husak, 2007) software was used to visualize residual electron density maps.

### Diphenyl [(R)-(+)-α-ethylbenzylamido]phosphate (I) Fractional atomic coordinates and isotropic or equivalent isotropic displacement parameters (Å^2^)

**Table:**

	*x*	*y*	*z*	*U*_iso_*/*U*_eq_	
P1	0.47169 (12)	0.71916 (8)	0.639453 (15)	0.0179	
O2	0.4682 (3)	0.7590 (2)	0.60181 (4)	0.0229	
C3	0.2732 (5)	0.8149 (3)	0.58334 (6)	0.0194	
C4	0.0936 (5)	0.9153 (3)	0.59535 (7)	0.0218	
C5	−0.0889 (5)	0.9701 (4)	0.57438 (7)	0.0264	
C6	−0.0868 (6)	0.9248 (4)	0.54188 (7)	0.0274	
C7	0.0950 (6)	0.8226 (4)	0.53038 (7)	0.0287	
C8	0.2758 (5)	0.7663 (4)	0.55103 (6)	0.0244	
O9	0.2348 (3)	0.6673 (2)	0.65240 (5)	0.0226	
O10	0.5773 (4)	0.8880 (2)	0.65292 (4)	0.0221	
C11	0.6063 (5)	0.9175 (3)	0.68651 (7)	0.0204	
C12	0.8179 (5)	0.8689 (3)	0.70180 (7)	0.0232	
C13	0.8488 (6)	0.9095 (4)	0.73416 (7)	0.0293	
C14	0.6729 (6)	0.9975 (4)	0.75073 (7)	0.0323	
C15	0.4628 (6)	1.0441 (4)	0.73500 (8)	0.0335	
C16	0.4291 (6)	1.0050 (4)	0.70255 (7)	0.0284	
N17	0.6906 (4)	0.5889 (3)	0.64476 (5)	0.0183	
C18	0.6554 (5)	0.4134 (3)	0.63655 (7)	0.0190	
C19	0.6338 (5)	0.3836 (3)	0.60013 (7)	0.0191	
C20	0.8049 (6)	0.4457 (3)	0.57846 (7)	0.0236	
C21	0.7840 (6)	0.4160 (4)	0.54532 (7)	0.0267	
C22	0.5918 (6)	0.3231 (3)	0.53328 (7)	0.0265	
C23	0.4216 (5)	0.2595 (4)	0.55459 (7)	0.0264	
C24	0.4427 (5)	0.2905 (3)	0.58786 (6)	0.0230	
C25	0.8638 (5)	0.3149 (3)	0.65162 (7)	0.0246	
C26	0.8837 (6)	0.3359 (4)	0.68815 (7)	0.0306	
H41	0.0953	0.9440	0.6176	0.0258*	
H51	−0.2169	1.0378	0.5825	0.0333*	
H61	−0.2126	0.9629	0.5284	0.0334*	
H71	0.0971	0.7921	0.5081	0.0351*	
H81	0.4006	0.6971	0.5432	0.0311*	
H121	0.9407	0.8103	0.6910	0.0282*	
H131	0.9901	0.8782	0.7449	0.0362*	
H141	0.6962	1.0245	0.7732	0.0385*	
H151	0.3406	1.1020	0.7466	0.0414*	
H161	0.2869	1.0386	0.6919	0.0336*	
H181	0.4995	0.3782	0.6465	0.0220*	
H201	0.9339	0.5088	0.5863	0.0278*	
H211	0.9021	0.4591	0.5309	0.0327*	
H221	0.5809	0.3042	0.5105	0.0308*	
H231	0.2889	0.1950	0.5464	0.0310*	
H241	0.3217	0.2488	0.6018	0.0291*	
H251	1.0183	0.3522	0.6420	0.0296*	
H252	0.8302	0.1979	0.6470	0.0306*	
H263	1.0151	0.2655	0.6967	0.0474*	
H262	0.9199	0.4496	0.6934	0.0469*	
H261	0.7302	0.3052	0.6981	0.0460*	
H171	0.837 (3)	0.625 (2)	0.6445 (7)	0.0219 (19)*	

### Diphenyl [(R)-(+)-α-ethylbenzylamido]phosphate (I) Atomic displacement parameters (Å^2^)

**Table:**

	*U*^11^	*U*^22^	*U*^33^	*U*^12^	*U*^13^	*U*^23^
P1	0.0183 (3)	0.0169 (3)	0.0184 (3)	0.0003 (3)	0.0003 (3)	−0.0009 (3)
O2	0.0178 (9)	0.0304 (10)	0.0207 (9)	0.0051 (9)	0.0004 (8)	0.0012 (7)
C3	0.0166 (13)	0.0204 (14)	0.0212 (13)	0.0002 (11)	−0.0014 (10)	0.0027 (10)
C4	0.0241 (15)	0.0203 (13)	0.0209 (13)	−0.0005 (12)	0.0004 (12)	−0.0009 (11)
C5	0.0215 (15)	0.0214 (14)	0.0364 (16)	0.0047 (12)	0.0003 (13)	0.0024 (12)
C6	0.0241 (15)	0.0249 (15)	0.0331 (16)	−0.0028 (12)	−0.0096 (13)	0.0053 (12)
C7	0.0328 (17)	0.0320 (16)	0.0213 (14)	−0.0007 (13)	−0.0056 (12)	0.0009 (12)
C8	0.0246 (14)	0.0262 (15)	0.0224 (13)	0.0035 (12)	0.0018 (11)	0.0007 (11)
O9	0.0194 (10)	0.0225 (10)	0.0259 (9)	0.0001 (8)	0.0017 (8)	−0.0009 (8)
O10	0.0248 (10)	0.0183 (9)	0.0233 (9)	−0.0007 (8)	−0.0009 (8)	−0.0003 (8)
C11	0.0222 (14)	0.0154 (12)	0.0235 (13)	−0.0048 (11)	0.0023 (11)	−0.0024 (11)
C12	0.0244 (15)	0.0221 (14)	0.0232 (14)	−0.0012 (12)	0.0029 (12)	−0.0018 (11)
C13	0.0314 (16)	0.0315 (16)	0.0252 (15)	−0.0041 (14)	−0.0009 (13)	0.0024 (13)
C14	0.0430 (19)	0.0289 (16)	0.0251 (15)	−0.0109 (15)	0.0066 (14)	−0.0058 (13)
C15	0.0321 (17)	0.0298 (16)	0.0385 (17)	−0.0019 (14)	0.0098 (15)	−0.0117 (13)
C16	0.0239 (15)	0.0232 (14)	0.0380 (16)	0.0030 (13)	0.0011 (13)	−0.0046 (12)
N17	0.0173 (11)	0.0173 (11)	0.0204 (12)	−0.0029 (9)	−0.0010 (10)	−0.0020 (9)
C18	0.0209 (13)	0.0146 (12)	0.0215 (13)	−0.0010 (11)	0.0033 (11)	−0.0024 (11)
C19	0.0204 (13)	0.0138 (13)	0.0230 (13)	0.0030 (11)	0.0017 (11)	−0.0028 (10)
C20	0.0235 (15)	0.0211 (14)	0.0261 (14)	−0.0029 (12)	0.0015 (12)	−0.0018 (11)
C21	0.0282 (16)	0.0245 (15)	0.0275 (15)	0.0017 (13)	0.0069 (13)	0.0021 (12)
C22	0.0339 (17)	0.0265 (15)	0.0191 (13)	0.0081 (13)	−0.0017 (12)	−0.0018 (11)
C23	0.0232 (15)	0.0270 (15)	0.0288 (14)	0.0019 (12)	−0.0056 (12)	−0.0075 (12)
C24	0.0211 (13)	0.0225 (13)	0.0254 (13)	0.0020 (13)	0.0020 (11)	−0.0004 (12)
C25	0.0295 (15)	0.0183 (14)	0.0261 (14)	0.0034 (12)	−0.0001 (12)	0.0018 (11)
C26	0.0413 (18)	0.0226 (15)	0.0279 (15)	0.0024 (14)	−0.0047 (13)	0.0016 (12)

### Diphenyl [(R)-(+)-α-ethylbenzylamido]phosphate (I) Geometric parameters (Å, º)

**Table:**

P1—O2	1.5814 (18)	C15—C16	1.384 (4)
P1—O9	1.469 (2)	C15—H151	0.948
P1—O10	1.5924 (19)	C16—H161	0.937
P1—N17	1.619 (2)	N17—C18	1.482 (3)
O2—C3	1.390 (3)	N17—H171	0.854 (17)
C3—C4	1.373 (4)	C18—C19	1.522 (4)
C3—C8	1.386 (4)	C18—C25	1.530 (4)
C4—C5	1.396 (4)	C18—H181	0.991
C4—H41	0.945	C19—C20	1.391 (4)
C5—C6	1.386 (4)	C19—C24	1.390 (4)
C5—H51	0.954	C20—C21	1.388 (4)
C6—C7	1.383 (4)	C20—H201	0.933
C6—H61	0.939	C21—C22	1.390 (4)
C7—C8	1.385 (4)	C21—H211	0.946
C7—H71	0.948	C22—C23	1.383 (4)
C8—H81	0.945	C22—H221	0.953
O10—C11	1.411 (3)	C23—C24	1.396 (4)
C11—C12	1.380 (4)	C23—H231	0.960
C11—C16	1.375 (4)	C24—H241	0.940
C12—C13	1.382 (4)	C25—C26	1.516 (4)
C12—H121	0.938	C25—H251	0.985
C13—C14	1.383 (5)	C25—H252	0.989
C13—H131	0.929	C26—H263	0.987
C14—C15	1.377 (5)	C26—H262	0.972
C14—H141	0.959	C26—H261	0.970
			
O2—P1—O9	113.77 (11)	C15—C16—H161	119.7
O2—P1—O10	99.62 (10)	C11—C16—H161	121.2
O9—P1—O10	116.45 (11)	P1—N17—C18	120.34 (18)
O2—P1—N17	106.00 (11)	P1—N17—H171	118.2 (12)
O9—P1—N17	114.90 (12)	C18—N17—H171	116.6 (12)
O10—P1—N17	104.43 (11)	N17—C18—C19	112.8 (2)
P1—O2—C3	127.68 (17)	N17—C18—C25	108.4 (2)
O2—C3—C4	123.5 (2)	C19—C18—C25	111.9 (2)
O2—C3—C8	114.9 (2)	N17—C18—H181	107.4
C4—C3—C8	121.5 (3)	C19—C18—H181	107.0
C3—C4—C5	119.0 (3)	C25—C18—H181	109.2
C3—C4—H41	119.3	C18—C19—C20	121.3 (2)
C5—C4—H41	121.7	C18—C19—C24	120.2 (2)
C4—C5—C6	120.3 (3)	C20—C19—C24	118.5 (3)
C4—C5—H51	120.0	C19—C20—C21	120.6 (3)
C6—C5—H51	119.7	C19—C20—H201	119.6
C5—C6—C7	119.7 (3)	C21—C20—H201	119.8
C5—C6—H61	118.4	C20—C21—C22	120.5 (3)
C7—C6—H61	121.9	C20—C21—H211	119.5
C6—C7—C8	120.5 (3)	C22—C21—H211	120.1
C6—C7—H71	119.7	C21—C22—C23	119.5 (3)
C8—C7—H71	119.7	C21—C22—H221	119.1
C3—C8—C7	119.0 (3)	C23—C22—H221	121.4
C3—C8—H81	120.3	C22—C23—C24	119.8 (3)
C7—C8—H81	120.7	C22—C23—H231	119.7
P1—O10—C11	121.91 (16)	C24—C23—H231	120.5
O10—C11—C12	119.5 (2)	C23—C24—C19	121.1 (3)
O10—C11—C16	118.5 (3)	C23—C24—H241	118.1
C12—C11—C16	121.8 (3)	C19—C24—H241	120.7
C11—C12—C13	118.2 (3)	C18—C25—C26	113.3 (2)
C11—C12—H121	122.5	C18—C25—H251	108.7
C13—C12—H121	119.3	C26—C25—H251	107.6
C12—C13—C14	120.9 (3)	C18—C25—H252	106.8
C12—C13—H131	119.6	C26—C25—H252	108.1
C14—C13—H131	119.5	H251—C25—H252	112.4
C13—C14—C15	119.8 (3)	C25—C26—H263	110.0
C13—C14—H141	120.0	C25—C26—H262	109.9
C15—C14—H141	120.1	H263—C26—H262	109.0
C14—C15—C16	120.1 (3)	C25—C26—H261	109.0
C14—C15—H151	119.7	H263—C26—H261	109.6
C16—C15—H151	120.2	H262—C26—H261	109.3
C15—C16—C11	119.1 (3)		

### Diphenyl [(R)-(+)-α-ethylbenzylamido]phosphate (I) Hydrogen-bond geometry (Å, º)

**Table:**

*D*—H···*A*	*D*—H	H···*A*	*D*···*A*	*D*—H···*A*
C12—H121···O9^i^	0.94	2.55	3.474 (4)	170
N17—H171···O9^i^	0.85	2.24	3.074 (4)	166 (2)

Symmetry code: (i) *x*+1, *y*, *z*.

### N-[(R)-(+)-α-Ethylbenzyl]-P,P-diphenylphosphinic amide (II) Crystal data

**Table:**

C_21_H_22_NOP	*Z* = 2
*M**_r_* = 335.39	*F*(000) = 356
Triclinic, *P*1	*D*_x_ = 1.233 Mg m^−^^3^
*a* = 9.0483 (7) Å	Cu *K*α radiation, λ = 1.54180 Å
*b* = 10.5533 (8) Å	Cell parameters from 11237 reflections
*c* = 11.0036 (6) Å	θ = 4–74°
α = 70.065 (6)°	µ = 1.39 mm^−^^1^
β = 86.368 (5)°	*T* = 95 K
γ = 66.571 (7)°	Needle, colorless
*V* = 903.15 (13) Å^3^	0.62 × 0.09 × 0.07 mm

### N-[(R)-(+)-α-Ethylbenzyl]-P,P-diphenylphosphinic amide (II) Data collection

**Table:**

Oxford Diffraction SuperNova diffractometer	6648 reflections with *I* > 2.0σ(*I*)
Focussing mirrors monochromator	*R*_int_ = 0.036
ω scans	θ_max_ = 74.8°, θ_min_ = 4.3°
Absorption correction: multi-scan (CrysAlisPro; Rigaku OD, 2017)	*h* = −10→11
*T*_min_ = 0.49, *T*_max_ = 0.91	*k* = −13→13
15035 measured reflections	*l* = −13→13
6781 independent reflections	

### N-[(R)-(+)-α-Ethylbenzyl]-P,P-diphenylphosphinic amide (II) Refinement

**Table:**

Refinement on *F*^2^	H atoms treated by a mixture of independent and constrained refinement
Least-squares matrix: full	Method = Modified Sheldrick *w* = 1/[σ^2^(*F*^2^) + ( 0.05*P*)^2^ + 0.53*P*] , where *P* = (max(*F*_o_^2^,0) + 2*F*_c_^2^)/3
*R*[*F* > 3σ(*F*)] = 0.034	(Δ/σ)_max_ = 0.001
*wR*(*F*) = 0.092	Δρ_max_ = 0.49 e Å^−^^3^
*S* = 0.97	Δρ_min_ = −0.40 e Å^−^^3^
6779 reflections	Extinction correction: Larson (1970), Equation 22
443 parameters	Extinction coefficient: 12 (2)
11 restraints	Absolute structure: Parsons *et al.* (2013), 3140 Friedel pairs
Primary atom site location: other	Absolute structure parameter: −0.013 (7)
Hydrogen site location: difference Fourier map	

### N-[(R)-(+)-α-Ethylbenzyl]-P,P-diphenylphosphinic amide (II) Special details

**Table:**

Experimental. The crystal was placed in the cold stream of an Oxford Cryosystems open-flow nitrogen cryostat (Cosier & Glazer, 1986) with a nominal stability of 0.1K. Cosier, J. & Glazer, A.M., 1986. J. Appl. Cryst. 105-107.

### N-[(R)-(+)-α-Ethylbenzyl]-P,P-diphenylphosphinic amide (II) Fractional atomic coordinates and isotropic or equivalent isotropic displacement parameters (Å^2^)

**Table:**

	*x*	*y*	*z*	*U*_iso_*/*U*_eq_	
P1	0.65963 (7)	0.68073 (7)	0.14489 (6)	0.0147	
O2	0.68651 (18)	0.78920 (16)	0.18817 (15)	0.0192	
N3	0.5062 (2)	0.7351 (2)	0.04120 (17)	0.0183	
C4	0.4906 (3)	0.8436 (2)	−0.0895 (2)	0.0186	
C5	0.4419 (3)	0.7964 (2)	−0.1908 (2)	0.0200	
C6	0.3292 (3)	0.7336 (2)	−0.1687 (2)	0.0246	
C7	0.2863 (3)	0.6919 (3)	−0.2628 (3)	0.0311	
C8	0.3541 (3)	0.7134 (3)	−0.3805 (3)	0.0375	
C9	0.4638 (3)	0.7781 (3)	−0.4047 (3)	0.0358	
C10	0.5082 (3)	0.8189 (3)	−0.3100 (2)	0.0280	
C11	0.3751 (3)	0.9980 (2)	−0.0941 (2)	0.0224	
C12	0.3668 (3)	1.1186 (3)	−0.2214 (2)	0.0301	
C13	0.6291 (3)	0.5395 (2)	0.2788 (2)	0.0171	
C14	0.5864 (3)	0.4341 (2)	0.2599 (2)	0.0231	
C15	0.5756 (3)	0.3206 (3)	0.3661 (3)	0.0283	
C16	0.6061 (3)	0.3133 (3)	0.4908 (2)	0.0326	
C17	0.6464 (3)	0.4184 (3)	0.5101 (2)	0.0339	
C18	0.6596 (3)	0.5315 (3)	0.4045 (2)	0.0244	
C19	0.8355 (3)	0.5873 (2)	0.0723 (2)	0.0163	
C20	0.9871 (3)	0.5761 (2)	0.1080 (2)	0.0228	
C21	1.1249 (3)	0.4987 (3)	0.0594 (3)	0.0291	
C22	1.1132 (3)	0.4300 (3)	−0.0238 (3)	0.0370	
C23	0.9630 (4)	0.4415 (3)	−0.0610 (3)	0.0366	
C24	0.8248 (3)	0.5197 (3)	−0.0129 (2)	0.0266	
P25	0.07166 (8)	0.86743 (7)	0.21332 (6)	0.0153	
O26	0.19971 (18)	0.79311 (17)	0.13860 (15)	0.0191	
N27	−0.1158 (2)	0.9595 (2)	0.14867 (17)	0.0186	
C28	−0.1622 (3)	1.0887 (2)	0.0291 (2)	0.0190	
C29	−0.1193 (3)	1.0513 (2)	−0.0942 (2)	0.0181	
C30	−0.0560 (3)	1.1317 (3)	−0.1945 (2)	0.0246	
C31	−0.0237 (3)	1.1016 (3)	−0.3093 (2)	0.0284	
C32	−0.0532 (3)	0.9893 (3)	−0.3247 (2)	0.0273	
C33	−0.1146 (3)	0.9065 (3)	−0.2249 (2)	0.0255	
C34	−0.1478 (3)	0.9383 (2)	−0.1108 (2)	0.0217	
C35	−0.3445 (3)	1.1792 (2)	0.0259 (2)	0.0236	
C36	−0.4123 (3)	1.3159 (3)	−0.0958 (3)	0.0292	
C37	0.0538 (3)	0.7326 (2)	0.3613 (2)	0.0170	
C38	0.1730 (3)	0.5907 (2)	0.3955 (2)	0.0199	
C39	0.1665 (3)	0.4812 (3)	0.5077 (2)	0.0262	
C40	0.0416 (3)	0.5138 (3)	0.5867 (2)	0.0262	
C41	−0.0766 (3)	0.6557 (3)	0.5537 (2)	0.0258	
C42	−0.0719 (3)	0.7651 (2)	0.4412 (2)	0.0216	
C43	0.1205 (3)	0.9967 (2)	0.2565 (2)	0.0189	
C44	0.2809 (3)	0.9847 (2)	0.2533 (2)	0.0212	
C45	0.3232 (3)	1.0805 (3)	0.2895 (2)	0.0264	
C46	0.2078 (3)	1.1884 (3)	0.3291 (2)	0.0277	
C47	0.0476 (3)	1.2019 (2)	0.3324 (2)	0.0243	
C48	0.0055 (3)	1.1064 (2)	0.2959 (2)	0.0209	
H41	0.5974	0.8444	−0.1091	0.0220*	
H61	0.2795	0.7205	−0.0895	0.0305*	
H71	0.2113	0.6464	−0.2449	0.0358*	
H81	0.3236	0.6870	−0.4464	0.0453*	
H91	0.5092	0.7947	−0.4848	0.0425*	
H101	0.5834	0.8620	−0.3268	0.0343*	
H111	0.2680	0.9967	−0.0769	0.0261*	
H112	0.4090	1.0174	−0.0231	0.0257*	
H121	0.2982	1.2149	−0.2168	0.0438*	
H122	0.3213	1.1064	−0.2928	0.0436*	
H123	0.4734	1.1161	−0.2430	0.0437*	
H141	0.5650	0.4389	0.1758	0.0280*	
H151	0.5505	0.2486	0.3538	0.0328*	
H161	0.6024	0.2356	0.5624	0.0404*	
H171	0.6628	0.4139	0.5962	0.0414*	
H181	0.6884	0.6026	0.4173	0.0270*	
H201	0.9926	0.6245	0.1670	0.0274*	
H211	1.2268	0.4921	0.0852	0.0351*	
H221	1.2068	0.3769	−0.0554	0.0433*	
H231	0.9537	0.3961	−0.1187	0.0442*	
H241	0.7251	0.5273	−0.0381	0.0319*	
H281	−0.1033	1.1485	0.0335	0.0213*	
H301	−0.0382	1.2104	−0.1855	0.0287*	
H311	0.0176	1.1593	−0.3771	0.0337*	
H321	−0.0304	0.9685	−0.4040	0.0327*	
H331	−0.1283	0.8249	−0.2332	0.0317*	
H341	−0.1856	0.8793	−0.0420	0.0267*	
H352	−0.4011	1.1147	0.0318	0.0272*	
H351	−0.3627	1.2111	0.1016	0.0271*	
H362	−0.5227	1.3761	−0.0867	0.0432*	
H361	−0.4124	1.2855	−0.1701	0.0425*	
H363	−0.3466	1.3730	−0.1133	0.0432*	
H381	0.2588	0.5680	0.3428	0.0238*	
H391	0.2475	0.3849	0.5279	0.0309*	
H401	0.0361	0.4387	0.6628	0.0323*	
H411	−0.1604	0.6800	0.6071	0.0306*	
H421	−0.1508	0.8603	0.4175	0.0247*	
H441	0.3606	0.9101	0.2267	0.0238*	
H451	0.4286	1.0731	0.2856	0.0318*	
H461	0.2360	1.2529	0.3533	0.0341*	
H471	−0.0304	1.2756	0.3581	0.0302*	
H481	−0.1012	1.1142	0.2988	0.0256*	
H271	−0.169 (2)	0.907 (2)	0.159 (2)	0.0223 (19)*	
H31	0.417 (2)	0.746 (2)	0.0763 (17)	0.0229 (19)*	

### N-[(R)-(+)-α-Ethylbenzyl]-P,P-diphenylphosphinic amide (II) Atomic displacement parameters (Å^2^)

**Table:**

	*U*^11^	*U*^22^	*U*^33^	*U*^12^	*U*^13^	*U*^23^
P1	0.0160 (2)	0.0161 (2)	0.0131 (2)	−0.0080 (2)	0.00316 (19)	−0.00467 (19)
O2	0.0189 (7)	0.0201 (7)	0.0238 (8)	−0.0108 (6)	0.0052 (6)	−0.0110 (6)
N3	0.0187 (9)	0.0216 (9)	0.0146 (8)	−0.0111 (7)	0.0048 (7)	−0.0032 (7)
C4	0.0205 (11)	0.0233 (10)	0.0133 (10)	−0.0131 (9)	0.0033 (8)	−0.0029 (8)
C5	0.0182 (10)	0.0200 (10)	0.0175 (10)	−0.0056 (8)	−0.0008 (8)	−0.0034 (8)
C6	0.0256 (12)	0.0231 (11)	0.0242 (11)	−0.0097 (10)	−0.0016 (9)	−0.0064 (9)
C7	0.0285 (13)	0.0254 (12)	0.0378 (14)	−0.0069 (10)	−0.0096 (11)	−0.0114 (11)
C8	0.0388 (16)	0.0336 (14)	0.0335 (14)	−0.0003 (12)	−0.0143 (12)	−0.0176 (12)
C9	0.0352 (15)	0.0427 (15)	0.0233 (12)	−0.0044 (12)	−0.0001 (11)	−0.0170 (11)
C10	0.0258 (12)	0.0350 (13)	0.0194 (11)	−0.0093 (10)	0.0028 (9)	−0.0083 (10)
C11	0.0272 (12)	0.0213 (11)	0.0186 (11)	−0.0121 (9)	0.0014 (9)	−0.0040 (9)
C12	0.0397 (14)	0.0233 (12)	0.0225 (12)	−0.0141 (11)	−0.0003 (10)	0.0001 (10)
C13	0.0144 (10)	0.0177 (10)	0.0171 (10)	−0.0054 (8)	0.0031 (8)	−0.0051 (8)
C14	0.0205 (11)	0.0238 (11)	0.0248 (11)	−0.0097 (9)	0.0071 (9)	−0.0079 (9)
C15	0.0253 (12)	0.0183 (11)	0.0378 (14)	−0.0108 (9)	0.0103 (10)	−0.0042 (10)
C16	0.0266 (12)	0.0243 (12)	0.0279 (13)	−0.0050 (10)	0.0102 (10)	0.0060 (10)
C17	0.0347 (14)	0.0377 (14)	0.0159 (11)	−0.0096 (11)	0.0062 (10)	0.0002 (10)
C18	0.0272 (12)	0.0258 (11)	0.0187 (11)	−0.0090 (10)	0.0015 (9)	−0.0081 (9)
C19	0.0165 (10)	0.0156 (9)	0.0161 (10)	−0.0077 (8)	0.0061 (8)	−0.0042 (8)
C20	0.0228 (11)	0.0210 (11)	0.0228 (11)	−0.0086 (9)	0.0035 (9)	−0.0059 (9)
C21	0.0209 (12)	0.0276 (12)	0.0373 (14)	−0.0124 (10)	0.0089 (10)	−0.0073 (11)
C22	0.0331 (14)	0.0324 (14)	0.0502 (17)	−0.0149 (11)	0.0257 (13)	−0.0216 (13)
C23	0.0477 (17)	0.0424 (15)	0.0403 (15)	−0.0287 (13)	0.0258 (13)	−0.0298 (13)
C24	0.0326 (13)	0.0336 (13)	0.0270 (12)	−0.0223 (11)	0.0116 (10)	−0.0171 (10)
P25	0.0168 (3)	0.0172 (2)	0.0135 (2)	−0.0086 (2)	0.00278 (19)	−0.00540 (19)
O26	0.0208 (8)	0.0215 (7)	0.0173 (7)	−0.0112 (6)	0.0044 (6)	−0.0068 (6)
N27	0.0201 (9)	0.0207 (9)	0.0179 (9)	−0.0133 (8)	0.0021 (7)	−0.0043 (7)
C28	0.0222 (11)	0.0156 (10)	0.0183 (10)	−0.0091 (8)	−0.0003 (8)	−0.0026 (8)
C29	0.0162 (10)	0.0167 (9)	0.0192 (10)	−0.0068 (8)	−0.0006 (8)	−0.0030 (8)
C30	0.0288 (12)	0.0226 (11)	0.0233 (11)	−0.0142 (10)	0.0007 (10)	−0.0040 (9)
C31	0.0291 (12)	0.0342 (13)	0.0165 (10)	−0.0147 (11)	0.0007 (9)	0.0002 (9)
C32	0.0240 (12)	0.0327 (13)	0.0183 (11)	−0.0056 (10)	0.0000 (9)	−0.0073 (10)
C33	0.0259 (12)	0.0293 (12)	0.0250 (12)	−0.0125 (10)	0.0028 (10)	−0.0120 (10)
C34	0.0226 (11)	0.0250 (11)	0.0201 (11)	−0.0132 (9)	0.0042 (9)	−0.0069 (9)
C35	0.0237 (12)	0.0228 (11)	0.0259 (12)	−0.0096 (9)	0.0015 (9)	−0.0099 (9)
C36	0.0240 (12)	0.0218 (11)	0.0359 (14)	−0.0056 (10)	−0.0056 (10)	−0.0058 (10)
C37	0.0187 (10)	0.0215 (10)	0.0152 (9)	−0.0113 (8)	0.0000 (8)	−0.0075 (8)
C38	0.0201 (11)	0.0220 (11)	0.0181 (10)	−0.0091 (9)	0.0040 (8)	−0.0070 (9)
C39	0.0303 (13)	0.0199 (11)	0.0216 (11)	−0.0085 (10)	0.0028 (9)	−0.0012 (9)
C40	0.0331 (13)	0.0263 (12)	0.0170 (10)	−0.0155 (10)	0.0023 (10)	−0.0005 (9)
C41	0.0258 (12)	0.0318 (12)	0.0199 (11)	−0.0125 (10)	0.0078 (9)	−0.0087 (9)
C42	0.0230 (11)	0.0224 (11)	0.0200 (11)	−0.0096 (9)	0.0032 (9)	−0.0076 (9)
C43	0.0221 (11)	0.0182 (10)	0.0142 (9)	−0.0069 (8)	0.0026 (8)	−0.0047 (8)
C44	0.0200 (11)	0.0258 (11)	0.0198 (11)	−0.0115 (9)	0.0025 (9)	−0.0074 (9)
C45	0.0259 (12)	0.0289 (12)	0.0321 (13)	−0.0177 (10)	0.0013 (10)	−0.0117 (10)
C46	0.0379 (14)	0.0257 (12)	0.0261 (12)	−0.0186 (11)	−0.0003 (10)	−0.0093 (10)
C47	0.0296 (13)	0.0207 (11)	0.0238 (11)	−0.0089 (9)	0.0038 (9)	−0.0108 (9)
C48	0.0226 (11)	0.0220 (10)	0.0187 (10)	−0.0112 (9)	0.0035 (9)	−0.0055 (8)

### N-[(R)-(+)-α-Ethylbenzyl]-P,P-diphenylphosphinic amide (II) Geometric parameters (Å, º)

**Table:**

P1—O2	1.4846 (15)	P25—O26	1.4933 (15)
P1—N3	1.6367 (19)	P25—N27	1.6426 (19)
P1—C13	1.802 (2)	P25—C37	1.808 (2)
P1—C19	1.808 (2)	P25—C43	1.797 (2)
N3—C4	1.474 (2)	N27—C28	1.469 (3)
N3—H31	0.856 (16)	N27—H271	0.841 (16)
C4—C5	1.517 (3)	C28—C29	1.524 (3)
C4—C11	1.532 (3)	C28—C35	1.538 (3)
C4—H41	0.980	C28—H281	0.988
C5—C6	1.391 (3)	C29—C30	1.391 (3)
C5—C10	1.391 (3)	C29—C34	1.386 (3)
C6—C7	1.383 (3)	C30—C31	1.391 (3)
C6—H61	0.948	C30—H301	0.944
C7—C8	1.383 (4)	C31—C32	1.378 (4)
C7—H71	0.953	C31—H311	0.951
C8—C9	1.380 (4)	C32—C33	1.390 (3)
C8—H81	0.950	C32—H321	0.961
C9—C10	1.389 (4)	C33—C34	1.392 (3)
C9—H91	0.940	C33—H331	0.952
C10—H101	0.936	C34—H341	0.945
C11—C12	1.520 (3)	C35—C36	1.527 (3)
C11—H111	0.980	C35—H352	0.986
C11—H112	0.967	C35—H351	0.982
C12—H121	0.975	C36—H362	0.968
C12—H122	0.977	C36—H361	0.975
C12—H123	0.971	C36—H363	0.972
C13—C14	1.393 (3)	C37—C38	1.390 (3)
C13—C18	1.395 (3)	C37—C42	1.397 (3)
C14—C15	1.390 (3)	C38—C39	1.391 (3)
C14—H141	0.938	C38—H381	0.941
C15—C16	1.387 (4)	C39—C40	1.387 (3)
C15—H151	0.928	C39—H391	0.945
C16—C17	1.379 (4)	C40—C41	1.387 (3)
C16—H161	0.934	C40—H401	0.952
C17—C18	1.392 (3)	C41—C42	1.387 (3)
C17—H171	0.949	C41—H411	0.937
C18—H181	0.940	C42—H421	0.929
C19—C20	1.399 (3)	C43—C44	1.406 (3)
C19—C24	1.387 (3)	C43—C48	1.392 (3)
C20—C21	1.383 (3)	C44—C45	1.389 (3)
C20—H201	0.967	C44—H441	0.950
C21—C22	1.379 (4)	C45—C46	1.382 (4)
C21—H211	0.951	C45—H451	0.925
C22—C23	1.391 (4)	C46—C47	1.398 (4)
C22—H221	0.933	C46—H461	0.932
C23—C24	1.384 (3)	C47—C48	1.388 (3)
C23—H231	0.942	C47—H471	0.933
C24—H241	0.925	C48—H481	0.935
			
O2—P1—N3	119.94 (9)	O26—P25—N27	119.61 (9)
O2—P1—C13	111.80 (9)	O26—P25—C37	109.99 (9)
N3—P1—C13	102.43 (10)	N27—P25—C37	102.69 (10)
O2—P1—C19	110.23 (9)	O26—P25—C43	110.87 (9)
N3—P1—C19	105.15 (10)	N27—P25—C43	104.84 (10)
C13—P1—C19	106.21 (9)	C37—P25—C43	108.07 (10)
P1—N3—C4	120.91 (14)	P25—N27—C28	122.16 (15)
P1—N3—H31	113.3 (12)	P25—N27—H271	114.1 (12)
C4—N3—H31	114.3 (12)	C28—N27—H271	115.3 (12)
N3—C4—C5	110.46 (17)	N27—C28—C29	114.02 (17)
N3—C4—C11	110.64 (17)	N27—C28—C35	107.53 (17)
C5—C4—C11	113.02 (18)	C29—C28—C35	111.51 (18)
N3—C4—H41	108.1	N27—C28—H281	108.1
C5—C4—H41	106.1	C29—C28—H281	106.9
C11—C4—H41	108.3	C35—C28—H281	108.7
C4—C5—C6	121.51 (19)	C28—C29—C30	121.5 (2)
C4—C5—C10	119.7 (2)	C28—C29—C34	120.35 (19)
C6—C5—C10	118.8 (2)	C30—C29—C34	118.2 (2)
C5—C6—C7	120.4 (2)	C29—C30—C31	121.3 (2)
C5—C6—H61	120.3	C29—C30—H301	119.1
C7—C6—H61	119.3	C31—C30—H301	119.5
C6—C7—C8	120.3 (3)	C30—C31—C32	119.9 (2)
C6—C7—H71	119.1	C30—C31—H311	119.8
C8—C7—H71	120.5	C32—C31—H311	120.3
C7—C8—C9	119.8 (2)	C31—C32—C33	119.6 (2)
C7—C8—H81	120.9	C31—C32—H321	119.7
C9—C8—H81	119.2	C33—C32—H321	120.7
C8—C9—C10	120.0 (2)	C32—C33—C34	120.1 (2)
C8—C9—H91	120.5	C32—C33—H331	119.6
C10—C9—H91	119.5	C34—C33—H331	120.2
C5—C10—C9	120.6 (3)	C33—C34—C29	120.9 (2)
C5—C10—H101	120.0	C33—C34—H341	119.7
C9—C10—H101	119.4	C29—C34—H341	119.3
C4—C11—C12	113.76 (19)	C28—C35—C36	113.74 (19)
C4—C11—H111	107.7	C28—C35—H352	108.0
C12—C11—H111	110.6	C36—C35—H352	109.3
C4—C11—H112	108.3	C28—C35—H351	107.8
C12—C11—H112	109.8	C36—C35—H351	108.2
H111—C11—H112	106.4	H352—C35—H351	109.8
C11—C12—H121	110.4	C35—C36—H362	110.1
C11—C12—H122	110.6	C35—C36—H361	109.1
H121—C12—H122	108.0	H362—C36—H361	108.1
C11—C12—H123	111.5	C35—C36—H363	111.5
H121—C12—H123	109.3	H362—C36—H363	110.1
H122—C12—H123	106.9	H361—C36—H363	107.8
P1—C13—C14	121.84 (17)	P25—C37—C38	117.22 (16)
P1—C13—C18	118.28 (17)	P25—C37—C42	123.28 (17)
C14—C13—C18	119.8 (2)	C38—C37—C42	119.5 (2)
C13—C14—C15	120.0 (2)	C37—C38—C39	120.3 (2)
C13—C14—H141	120.4	C37—C38—H381	120.1
C15—C14—H141	119.6	C39—C38—H381	119.6
C14—C15—C16	119.9 (2)	C38—C39—C40	120.0 (2)
C14—C15—H151	120.2	C38—C39—H391	118.8
C16—C15—H151	119.9	C40—C39—H391	121.2
C15—C16—C17	120.3 (2)	C39—C40—C41	119.9 (2)
C15—C16—H161	120.3	C39—C40—H401	120.1
C17—C16—H161	119.4	C41—C40—H401	120.1
C16—C17—C18	120.2 (2)	C40—C41—C42	120.4 (2)
C16—C17—H171	119.2	C40—C41—H411	120.9
C18—C17—H171	120.5	C42—C41—H411	118.7
C13—C18—C17	119.7 (2)	C37—C42—C41	119.9 (2)
C13—C18—H181	119.8	C37—C42—H421	118.8
C17—C18—H181	120.5	C41—C42—H421	121.3
P1—C19—C20	119.48 (17)	P25—C43—C44	118.93 (17)
P1—C19—C24	121.36 (18)	P25—C43—C48	122.26 (18)
C20—C19—C24	119.1 (2)	C44—C43—C48	118.8 (2)
C19—C20—C21	120.8 (2)	C43—C44—C45	120.2 (2)
C19—C20—H201	118.2	C43—C44—H441	119.6
C21—C20—H201	121.0	C45—C44—H441	120.1
C20—C21—C22	119.6 (2)	C44—C45—C46	120.3 (2)
C20—C21—H211	119.4	C44—C45—H451	119.7
C22—C21—H211	120.9	C46—C45—H451	120.0
C21—C22—C23	120.1 (2)	C45—C46—C47	120.1 (2)
C21—C22—H221	119.2	C45—C46—H461	120.4
C23—C22—H221	120.7	C47—C46—H461	119.5
C22—C23—C24	120.3 (2)	C46—C47—C48	119.6 (2)
C22—C23—H231	120.7	C46—C47—H471	120.0
C24—C23—H231	119.1	C48—C47—H471	120.4
C19—C24—C23	120.1 (2)	C43—C48—C47	121.0 (2)
C19—C24—H241	119.9	C43—C48—H481	119.0
C23—C24—H241	120.0	C47—C48—H481	120.0

### N-[(R)-(+)-α-Ethylbenzyl]-P,P-diphenylphosphinic amide (II) Hydrogen-bond geometry (Å, º)

**Table:**

*D*—H···*A*	*D*—H	H···*A*	*D*···*A*	*D*—H···*A*
N27—H271···O2^i^	0.84	2.08	2.923 (4)	176 (2)
N3—H31···O26	0.86	1.97	2.817 (4)	173 (2)

Symmetry code: (i) *x*−1, *y*, *z*.
