# Supplementary material for: Diastereotopic groups in two new single-enanti­omer structures (R 2)P(O)[NH-(+)CH(C2H5)(C6H5)] (R = OC6H5 and C6H5)
Source: Acta Crystallogr E Crystallogr Commun. 2023 Aug 1;79(Pt 9):769–76. doi: 10.1107/S2056989023006278 (PMC10483558; doi:10.1107/S2056989023006278)
Supplement: Supplementary file 2 [file e-79-00769-sup3.docx]

Diastereotopic Groups in Two New Single-Enantiomer Structures (*R*_2_P(O)[NH-(+)CH(C_2_H_5_)(C_6_H_5_)], *R* = OC_6_H_5_ and C_6_H_5_)

**Farnaz Eslami^a^, Mehrdad Pourayoubi^b*^, Fahimeh Sabbaghi^c^, Eliška Skořepová^d^, Michal Dušek^d^, and Sahar Baniyaghoob^a^**

^a^Department of Chemistry, Science and Research Branch, Islamic Azad University, Tehran, Iran; ^b^Department of Chemistry, Faculty of Science, Ferdowsi University of Mashhad, Mashhad, Iran; ^c^Department of Chemistry, Zanjan Branch, Islamic Azad University, Zanjan, Iran; ^d^Institute of Physics of the Czech Academy of Sciences, Na Slovance 2, 182 21 Prague 8, Czech Republic

e-mail: pourayoubi@um.ac.ir

**Table S1.** The bond lengths of diastereotopic P—Y bonds in the structures with a CNP(X)Y_2_ fragment (X = O, S, N; C is a dissymmetric carbon atom) retrieved from the Cambridge Structural Database (CSD version 5.42, updated on Feb 2021). The data of structures with chiral space groups were given.

| Entry | Structure | Refcode | Lengths of diastereotopic P—O bonds (Å) | Skeleton |
| --- | --- | --- | --- | --- |
| 1 | 1 | XAKDEW-Moiety 1 | 1.574/1.591 | P(O)(N)(O)_2_ |
| 2 |  | XAKDEW-Moiety 2 | 1.580/1.596 |  |
| 3 | 2 | EXIQIM | 1.582/1.586 | P(O)(N)(O)_2_ |
| 4 | 3 | HAHXUM-Mol 1 | 1.574/1.575 | P(O)(N)(O)_2_ |
| 5 |  | HAHXUM-Mol 2 | 1.566/1.555 |  |
| 6 | 4 | KEZTEO | 1.607/1.595 | P(O)(N)(O)_2_ |
| 7 | 5 | PEQFAU | 1.572/1.572 | P(O)(N)(O)_2_ |
| Entry | Structure | Refcode | Lengths of diastereotopic P—C bonds (Å) | Skeleton |
| 1 | 1 | PACSEV | 1.809/1.805 | P(O)(N)(C)_2_ |
| 2 | 2 | ATUCOI-Mol 1 | 1.807/1.810 | P(O)(N)(C)_2_ |
| 3 |  | ATUCOI-Mol 2 | 1.815/1.801 |  |
| 4 |  | ATUCOI-Mol 3 | 1.807/1.802 |  |
| 5 |  | ATUCOI-Mol 4 | 1.792/1.807 |  |
| 6 | 3 | BITHOE | 1.810/1.810 | P(O)(N)(C)_2_ |
| 7 | 4 | EMUNIK | 1.799/1.810 | P(S)(N)(C)_2_ |
| 8 | 5 | EWIBOD | 1.796/1.794 | P(O)(N)(C)_2_ |
| 9 | 6 | FABPIK | 1.821/1.813 | P(S)(N)(C)_2_ |
| 10 | 7 | GUZGAJ | 1.801/1.795 | P(O)(N)(C)_2_ |
| 11 | 8 | LESMEC-Mol 1-Moiety 1 | 1.801/1.798 | P(O)(N)(C)_2_ |
| 12 |  | LESMEC-Mol 1-Moiety 2 | 1.796/1.797 |  |
| 13 |  | LESMEC-Mol 2-Moiety 1 | 1.809/1.798 |  |
| 14 |  | LESMEC-Mol 2-Moiety 2 | 1.802/1.794 |  |
| 15 | 9 | MEFCIK | 1.817/1.816 | P(O)(N)(C)_2_ |
| 16 | 10 | MEFPUL | 1.837/1.820 | P(N)(N)(C)_2_ |
| 17 | 11 | MOCPEA | 1.796/1.806 | P(O)(N)(C)_2_ |
| 18 | 12 | NIFDEN | 1.803/1.800 | P(O)(N)(C)_2_ |
| 19 | 13 | OLUDUW-Mol 1 | 1.792/1.798 | P(O)(N)(C)_2_ |
| 20 |  | OLUDUW-Mol 2 | 1.802/1.804 |  |
| 21 |  | OLUDUW-Mol 3 | 1.792/1.802 |  |
| 22 | 14 | OLUFAE-Mol 1 | 1.806/1.812 | P(O)(N)(C)_2_ |
| 23 |  | OLUFAE-Mol 2 | 1.806/1.801 |  |
| 24 |  | OLUFAE-Mol 3 | 1.814/1.808 |  |
| 25 |  | OLUFAE-Mol 4 | 1.813/1.810 |  |
| 26 | 15 | OLUFEI | 1.808/1.815 | P(O)(N)(C)_2_ |
| 27 | 16 | OLUMIT-Mol 1 | 1.807/1.808 | P(O)(N)(C)_2_ |
| 28 |  | OLUMIT-Mol 2 | 1.808/1.809 |  |
| 29 | 17 | OMIGAS | 1.798/1.806 | P(S)(N)(C)_2_ |
| 30 | 18 | OVOCUZ-Mol 1 | 1.800/1.807 | P(O)(N)(C)_2_ |
| 31 |  | OVOCUZ-Mol 2 | 1.808/1.802 |  |
| 32 | 19 | POPQUH-Mol 1 | 1.814/1.815 | P(S)(N)(C)_2_ |
| 33 |  | POPQUH-Mol 2 | 1.811/1.813 |  |
| 34 | 20 | QICNIZ-Moiety 1 | 1.805/1.810 | P(S)(N)(C)_2_ |
| 35 |  | QICNIZ-Moiety 2 | 1.812/1.820 |  |
| 36 | 21 | QOMJUY | 1.818/1.804 | P(S)(N)(C)_2_ |
| 37 | 22 | RAPGIY | 1.773/1.778 | P(O)(N)(C)_2_ |
| 38 | 23 | RIDNAW | 1.796/1.809 | P(O)(N)(C)_2_ |
| 39 | 24 | RIDNEA | 1.804/1.813 | P(O)(N)(C)_2_ |
| 40 | 25 | RILSEN-Mol 1 | 1.802/1.809 | P(O)(N)(C)_2_ |
| 41 |  | RILSEN-Mol 2 | 1.810/1.802 |  |
| 42 | 26 | ROGHED | 1.782/1.782 | P(O)(N)(C)_2_ |
| 43 | 27 | ROJYAT-Mol 1 | 1.821/1.805 | P(O)(N)(C)_2_ |
| 44 |  | ROJYAT-Mol 2 | 1.807/1.807 |  |
| 45 |  | ROJYAT-Mol 3 | 1.819/1.804 |  |
| 46 |  | ROJYAT-Mol 4 | 1.807/1.819 |  |
| 47 | 28 | SURYEK-Mol 1 | 1.810/1.808 | P(O)(N)(C)_2_ |
| 48 |  | SURYEK-Mol 2 | 1.809/1.800 |  |
| 49 |  | SURYEK-Mol 3 | 1.787/1.808 |  |
| 50 |  | SURYEK-Mol 4 | 1.798/1.795 |  |
| 51 |  | SURYEK-Mol 5 | 1.798/1.812 |  |
| 52 |  | SURYEK-Mol 6 | 1.803/1.814 |  |
| 53 |  | SURYEK-Mol 7 | 1.809/1.802 |  |
| 54 |  | SURYEK-Mol 8 | 1.805/1.803 |  |
| 55 | 29 | TEFQEC-Mol 1 | 1.792/1.802 | P(O)(N)(C)_2_ |
| 56 |  | TEFQEC-Mol 2 | 1.804/1.802 |  |
| 57 | 30 | TEGBIS | 1.801/1.790 | P(O)(N)(C)_2_ |
| 58 | 31 | UCUCEC | 1.813/1.821 | P(S)(N)(C)_2_ |
| 59 | 32 | VUGSOG | 1.799/1.799 | P(O)(N)(C)_2_ |
| 60 | 33 | XIWZAF-Mol 1 | 1.819/1.809 | P(O)(N)(C)_2_ |
| 61 |  | XIWZAF-Mol 2 | 1.805/1.816 |  |
| 62 | 34 | YOHYUQ-Mol 1 | 1.787/1.812 | P(O)(N)(C)_2_ |
| 63 |  | YOHYUQ-Mol 2 | 1.792/1.801 |  |
| 64 | 35 | YOHZAX | 1.817/1.817 | P(O)(N)(C)_2_ |
| 65 | 36 | YOVBIX | 1.805/1.798 | P(O)(N)(C)_2_ |
| 66 | 37 | ZUGHAN | 1.802/1.797 | P(O)(N)(C)_2_ |
| 67 | 38 | ZUGHUH | 1.803/1.800 | P(O)(N)(C)_2_ |
| Entry | Structure | Refcode | Lengths of diastereotopic P—N bonds (Å) | Skeleton |
| 1 | 1 | FECHUT | 1.652/1.644 | P(O)(N)(N)_2_ |
| 2 | 2 | SOZREG-Mol1 | 1.634/1.646 | P(O)(N)(N)_2_ |
| 3 |  | SOZREG-Mol2 | 1.629/1.644 |  |
| 4 | 3 | SOZRIK-Mol 1 | 1.643/1.643 | P(O)(N)(N)_2_ |
| 5 |  | SOZRIK-Mol 2 | 1.643/1.650 |  |

**Table S2.** Torsion angles (TAs) and conformations of the chiral structures with O_2_P(O) and C_2_P(X) (X = O, S, N) fragments. The superscripts “a” or “b” above “Entry” denote to O_2_P- and C_2_P-based structures, respectively.

| ^a^Entry | Structure | Refcode | TAs related to diastereotopic groups (°) | Conformations | Atoms in TA |
| --- | --- | --- | --- | --- | --- |
| 1 | 1 | XAKDEW-Moiety 1 | –53.44/131.12 and –20.47/161.25 | –sc+ac and –sp+ap | C—C—O—P |
| 2 |  | XAKDEW-Moiety 2 | –70.73/113.57 and 84.26/–99.95 | –sc+ac and sc–ac |  |
| 3 | 2 | EXIQIM | –32.57/149.53 and –86.76/98.48 | –sc+ac and –sc+ac | C—C—O—P |
| 4 | 3 | KEZTEO | –33.44/151.22 and 64.08/–118.23 | –sc+ap and sc–ac | C—C—O—P |
| ^b^Entry | Structure | Refcode | TAs related to diastereotopic groups (°) | Conformations | Atoms in TA |
| 1 | 1 | PACSEV | –4.44/177.46 and –15.53/167.96 | –sp+ap and –sp+ap | C—C—P═O |
| 2 | 2 | ATUCOI-Mol 1 | –12.95/169.55 and 9.33/–174.67 | –sp+ap and sp–ap | C—C—P═O |
| 3 |  | ATUCOI-Mol 2 | –21.5/161.32 and 6.12/–176.78 | –sp+ap and sp–ap |  |
| 4 |  | ATUCOI-Mol 3 | –12.34/170.15 and 12.28/–173.37 | –sp+ap and sp–ap |  |
| 5 |  | ATUCOI-Mol 4 | 9.44/–175.13 and –22.00/162.37 | sp–ap and –sp+ap |  |
| 6 | 3 | EMUNIK | –32.5/147.8 and –41.78/139.18 | –sc+ac and –sc+ac | C—C—P═S |
| 7 | 4 | EWIBOD | 20.49/–160.83 and –24.76/157.08 | sp–ap and –sp+ap | C—C—P═O |
| 8 | 5 | FABPIK | –2.68/175.54 and –76.64/101.83 | –sp+ap and –sc+ac | C—C—P═S |
| 9 | 6 | GUZGAJ | 4.36/–177.37 and 20.08/–159.73 | sp–ap and sp–ap | C—C—P═O |
| 10 | 7 | LESMEC-Mol 1-Moiety 1 | 21.05/–161.87 and 62.7/–111.06 | sp–ap and sc–ac | C—C—P═O |
| 11 |  | LESMEC-Mol 1-Moiety 2 | –8.4/173.25 and –67.29/104.11 | –sp+ap and –sc+ac |  |
| 12 |  | LESMEC-Mol 2-Moiety 1 | 0.99/–172.86 and 12.21/–172.46 | sp–ap and sp–ap |  |
| 13 |  | LESMEC-Mol 2-Moiety 2 | –2.99/172.17 and –21.15/165.63 | –sp+ap and –sp+ap |  |
| 14 | 8 | MEFCIK | 0.04/176.54 and –13.3/169.66 | sp+ap and –sp+ap | C—C—P═O |
| 15 | 9 | MEFPUL | –40.25/–172.12 and –60.06/64.06 | –sc–ap and –sc+sc | C—C—P═N |
| 16 | 10 | MOCPEA | –11.68/167.72 and 15.38/–166.34 | –sp+ap and sp–ap | C—C—P═O |
| 17 | 11 | NIFDEN | –17.41/163.67 and –53.5/122.48 | –sp+ap and –sc+ac | C—C—P═O |
| 18 | 12 | OLUDUW-Mol 1 | 38.14/–143.39 and 33.6/–143.81 | sc–ac and sc–ac | C—C—P═O |
| 19 |  | OLUDUW-Mol 2 | 10.7/–167.3 and –16.36/165.7 | sp–ap and –sp+ap |  |
| 20 |  | OLUDUW-Mol 3 | 20.27/–162.84 and 71.74/–103.82 | sp–ap and sc–ac |  |
| 21 | 13 | OLUFAE-Mol 1 | 5.22/–171.2 and –83.45/90.41 | sp–ap and –sc+ac | C—C—P═O |
| 22 |  | OLUFAE-Mol 2 | 4.69/–175.16 and 71.6/–102.07 | sp–ap and sc–ac |  |
| 23 |  | OLUFAE-Mol 3 | –10.26/170.18 and 23.95/–153.46 | –sp+ap and sp–ap |  |
| 24 |  | OLUFAE-Mol 4 | 29.34/–149.85 and 25.66/–157.55 | sp–ac and sp–ap |  |
| 25 | 14 | OLUFEI | –14.32/164.98 and –55.74/122.17 | –sp+ap and –sc+ac | C—C—P═O |
| 26 | 15 | OLUMIT-Mol 1 | 18.29/–165.98 and 71.37/–103.28 | sp–ap and sc–ac | C—C—P═O |
| 27 |  | OLUMIT-Mol 2 | 16.65/–168.51 and 70.97/–104.67 | sp–ap and sc–ac |  |
| 28 | 16 | OMIGAS | 0.73/–179.45 and 32.39/–151.42 | sp–ap and sc–ap | C—C—P═S |
| 29 | 17 | OVOCUZ-Mol 1 | –2.03/178.89 and 81.13/–94.44 | –sp+ap and sc–ac | C—C—P═O |
| 30 |  | OVOCUZ-Mol 2 | –6.15/171.49 and –74.34/98.82 | –sp+ap and –sc+ac |  |
| 31 | 18 | POPQUH-Mol 1 | 26.00/–156.34 and 63.23/–115.23 | sp–ap and sc–ac | C—C—P═S |
| 32 |  | POPQUH-Mol 2 | –25.98/151.05 and –22.35/162.17 | –sp+ap and –sp+ap |  |
| 33 | 19 | QICNIZ-Moiety 1 | 10.69/–169.28 and –15.43/170.09 | sp–ap and –sp+ap | C—C—P═S |
| 34 |  | QICNIZ-Moiety 2 | 12.44/–169.51 and –20.14/160.95 | sp–ap and –sp+ap |  |
| 35 | 20 | QOMJUY | 49.87/–125.63 and 52.62/–127.92 | sc–ac and sc–ac | C—C—P═S |
| 36 | 21 | RAPGIY | –30.79/147.98 and –41.19/143.19 | –sc+ac and –sc+ac | C—C—P═O |
| 37 | 22 | RIDNAW | –17.74/168.28 and –67.75/108.54 | –sp+ap and –sc+ac | C—C—P═O |
| 38 | 23 | RIDNEA | –17.72/169.13 and –63.01/113.27 | –sp+ap and –sc+ac | C—C—P═O |
| 39 | 24 | RILSEN-Mol 1 | 9.13/–167.93 and 64.72/–111.11 | sp–ap and sc–ac | C—C—P═O |
| 40 |  | RILSEN-Mol 2 | 13.08/–162.42 and –86.07/87.73 | sp–ap and –sc+sc |  |
| 41 | 25 | ROGHED | 17.82/–166.38 and 44.17/–133.52 | sp–ap and sc–ac | C—C—P═O |
| 42 | 26 | ROJYAT-Mol 1 | 39.51/–140.4 and –50.68/124.94 | sc–ac and –sc+ac | C—C—P═O |
| 43 |  | ROJYAT-Mol 2 | 40.15/–139.09 and –52.79/124.96 | sc–ac and –sc+ac |  |
| 44 |  | ROJYAT-Mol 3 | 41.85/–139.51 and –53.14/122.08 | sc–ac and –sc+ac |  |
| 45 |  | ROJYAT-Mol 4 | 39.9/–139.14 and –53.75/122.42 | sc–ac and –sc+ac |  |
| 46 | 27 | SURYEK-Mol 1 | 4.47/–175.91 and 7.07/–171.09 | sp–ap and sp–ap | C—C—P═O |
| 47 |  | SURYEK-Mol 2 | –9.03/171.82 and –10.83/170.13 | –sp+ap and –sp+ap |  |
| 48 |  | SURYEK-Mol 3 | –9.75/172.13 and 10.18/–173.77 | –sp+ap and sp–ap |  |
| 49 |  | SURYEK-Mol 4 | 6.63/–175.82 and –36.38/143.7 | sp–ap and –sc+ac |  |
| 50 |  | SURYEK-Mol 5 | 17.1/–169.28 and 87.61/–87.62 | sp–ap and sc–sc |  |
| 51 |  | SURYEK-Mol 6 | –6.41/174.88 and 11.6/–168.86 | –sp+ap and sp–ap |  |
| 52 |  | SURYEK-Mol 7 | –7.52/172.43 and 18.38/–161.94 | –sp+ap and sp–ap |  |
| 53 |  | SURYEK-Mol 8 | 8.76/–168.62 and –11.79/168.46 | sp–ap and –sp+ap |  |
| 54 | 28 | TEFQEC-Mol 1 | –11.47/162.65 and –56.16/121.64 | –sp+ap and –sc+ac | C—C—P═O |
| 55 |  | TEFQEC-Mol 2 | 2.4/–177.19 and –18.25/162.74 | sp–ap and –sp+ap |  |
| 56 | 29 | TEGBIS | –5.39/170.59 and –21.83/160.24 | –sp+ap and –sp+ap | C—C—P═O |
| 57 | 30 | UCUCEC | 0.85/178.9 and –30.28/154.17 | sp+ap and –sc+ap | C—C—P═S |
| 58 | 31 | VUGSOG | –6.78/177.18 and –67.39/105.43 | –sp+ap and –sc+ac | C—C—P═O |
| 59 | 32 | YOHYUQ-Mol 1 | –8.18/171.99 and 74.25/–101.01 | –sp+ap and sc–ac | C—C—P═O |
| 60 |  | YOHYUQ-Mol 2 | –7.84/174.34 and 73.91/–99.75 | –sp+ap and sc–ac |  |
| 61 | 33 | YOHZAX | 35.72/–138.28 and 44.12/–136.81 | sc–ac and sc–ac | C—C—P═O |
| 62 | 34 | YOVBIX | 13.6/–165.71 and –5.27/175.69 | sp–ap and –sp+ap | C—C—P═O |
| 63 | 35 | ZUGHAN | 13.74/–167.81 and –8.14/173.49 | sp–ap and –sp+ap | C—C—P═O |
| 64 | 36 | ZUGHUH | –3.49/176.62 and 68.25/–107.82 | –sp+ap and sc–ac | C—C—P═O |


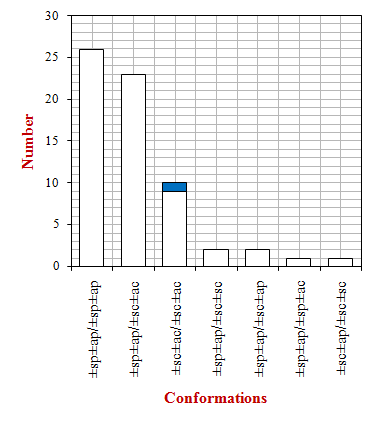


**Figure S1.** The conformations of diastereotopic groups (separated by “/”) in the C_2_P(X)-based structures (X = O, S, N) retrieved from the CSD (excluding metal complexes and compounds with a phosphorus-containing heterocyclic segment). The blue color shows the conformations of diastereotopic groups in the structure **II**.

**NMR spectroscopy**

In ^31^P NMR, the phosphorus signals of amidophosphodiester **I** and phosphinic amide **II** appear at –2.16 and 21.13 ppm, respectively. The high difference of phosphorus chemical shifts is related to different anisotropic effects of phenyl rings on phosphorus atoms. In **I**, the phosphorus was separated by an ester oxygen atom from the corresponding ring and in **II**, the phosphorus is directly bonded to the rings. This difference causes to displacing the phosphorus atoms in the zones where respectively more and less magnetic fields are needed, as observed in analogous compounds (Vahdani Alviri *et al*., 2020; Hamzehee *et al*., 2017).

In the ^1^H NMR, the N—H protons of **I** and **II** appear as triplets at 3.84 ppm (*J* = 10.8 Hz) and 5.91 ppm (*J* = 10.2 Hz), respectively, due to geminal phosphorus-hydrogen and vicinal hydrogen-hydrogen couplings. The two C_6_H_5_O groups in **I** and two C_6_H_5_ groups in **II** bonded to phosphorus are diastereotopic, and two sets of peaks are observed for them in the aromatic region of both ^1^H and ^13^C NMR spectra, and in the ^1^H NMR spectra, the related signals overlap with each other and with those of the chiral amine.

The assignments of ^13^C NMR spectra (discussed in the paper) were confirmed with DEPT experiments. The three doublets of **I**, at 143.04 ppm, and 150.74/150.92 ppm, vanish in the DEPT experiment and belong to the *ipso*-carbon atoms with three- and two-bond separations from phosphorus nucleus, respectively. For **II**, the doublet signals at 145.50 and 134.44/134.77 ppm vanish, showing the *ipso*-carbon atoms of chiral amine and diastereotopic phenyl groups (Figures S2 to S11).


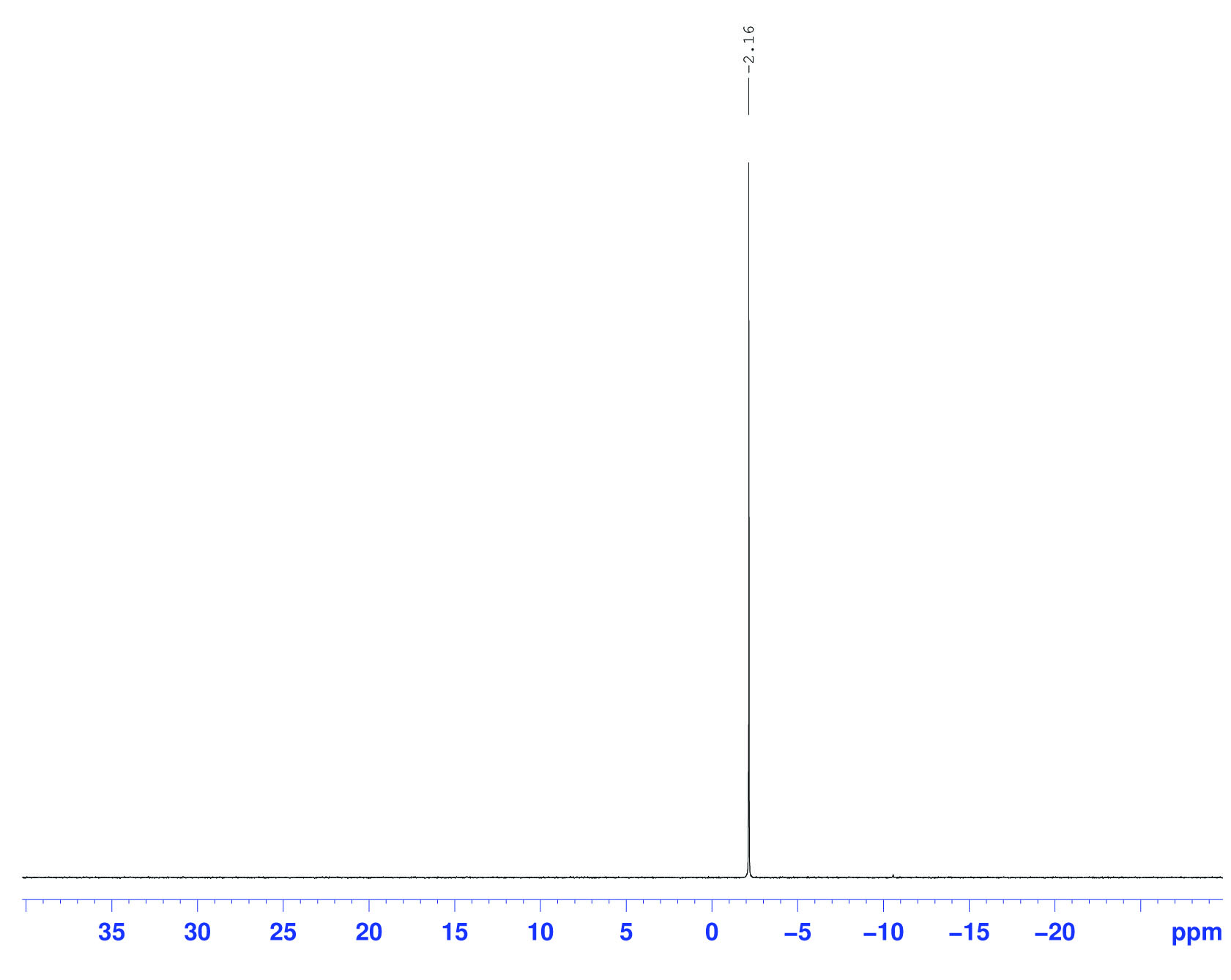


**Figure S2.** ^31^P{^1^H} NMR spectrum of **I** (in CDCl_3_).


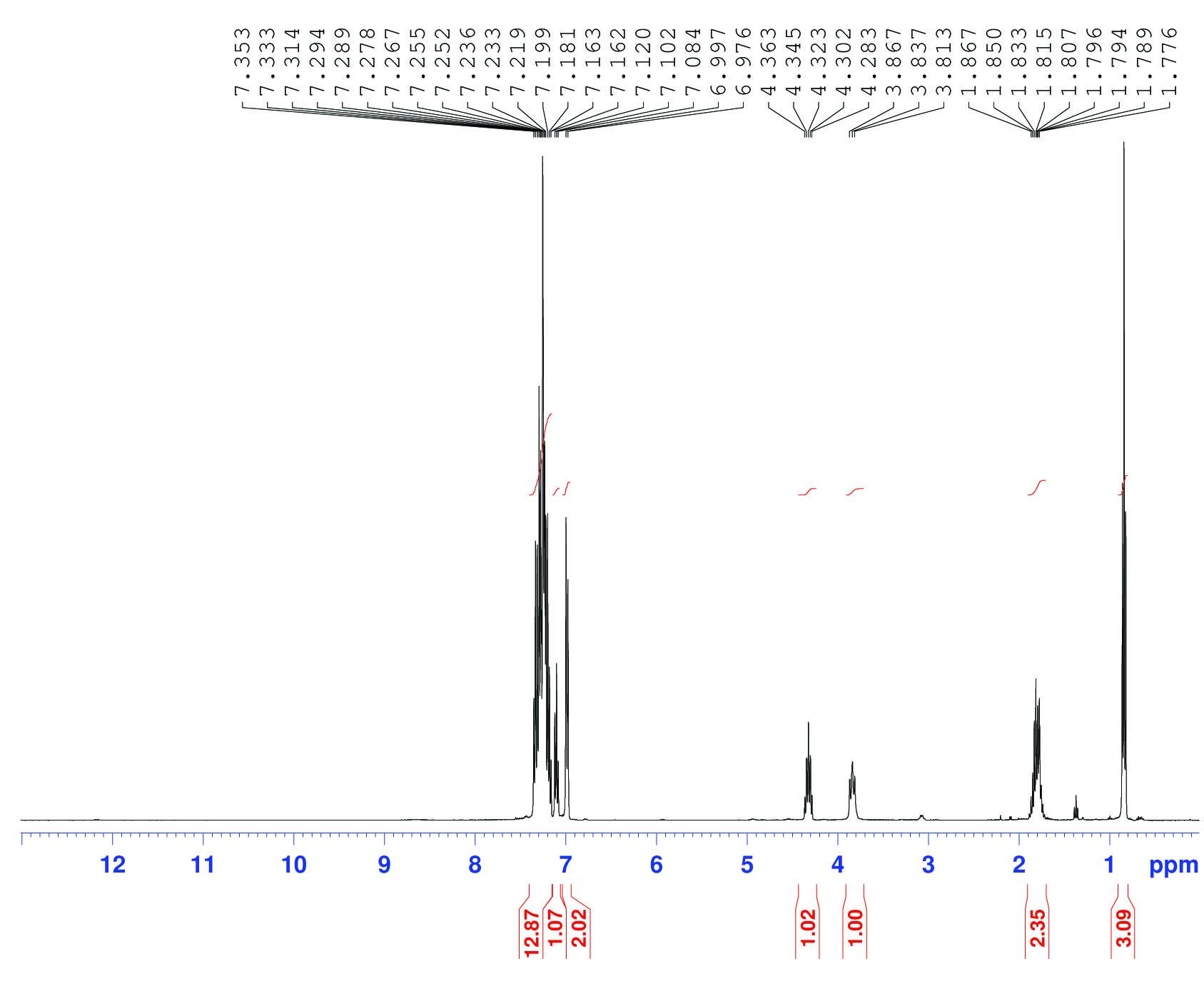


**Figure S3.** ^1^H NMR spectrum of **I** (in CDCl_3_).


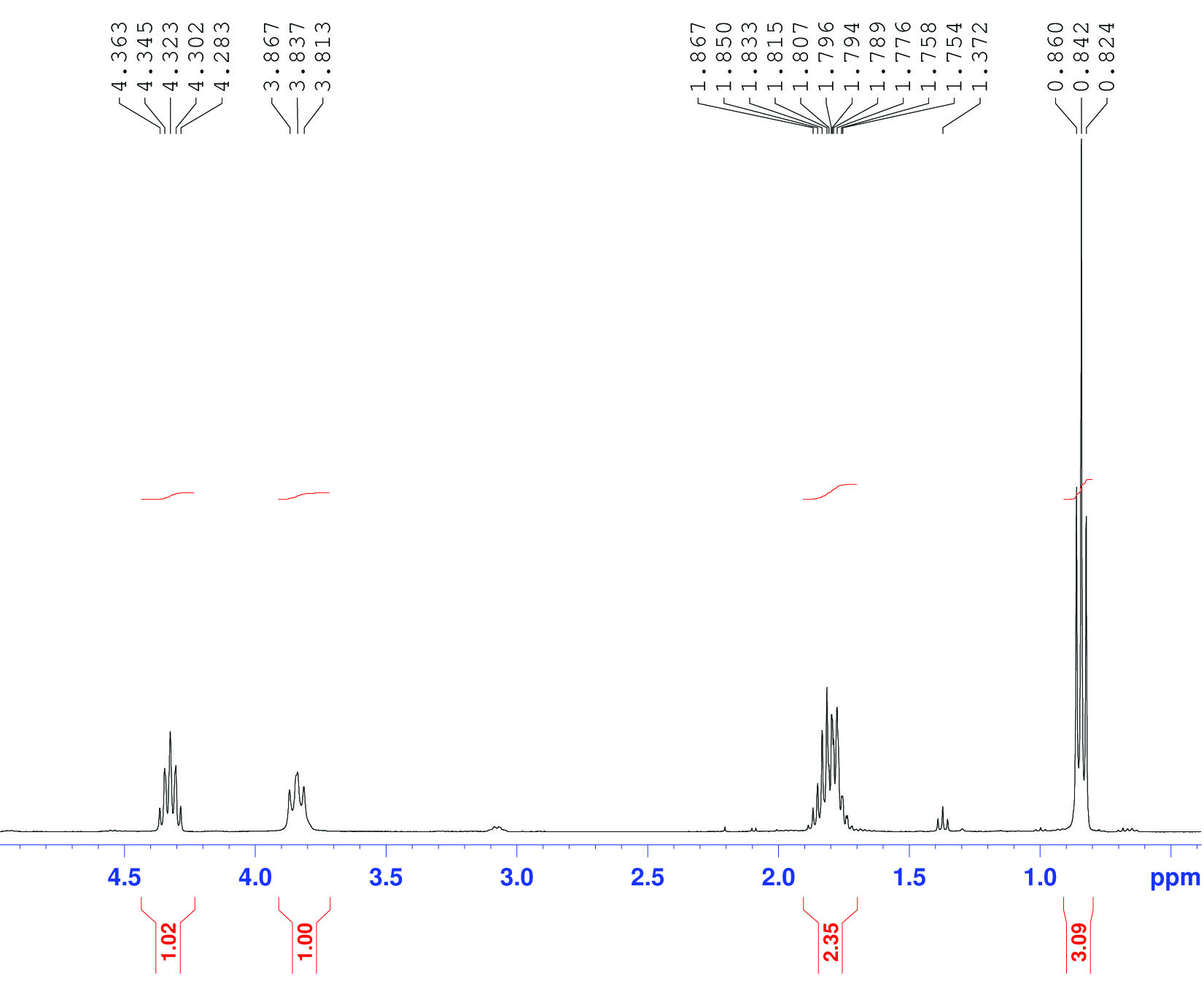


**Figure S4.** ^1^H NMR spectrum of **I** in the range of 0 to 4.5 ppm (the signals are related to CH_3_, CH_2_, NH and CH groups).


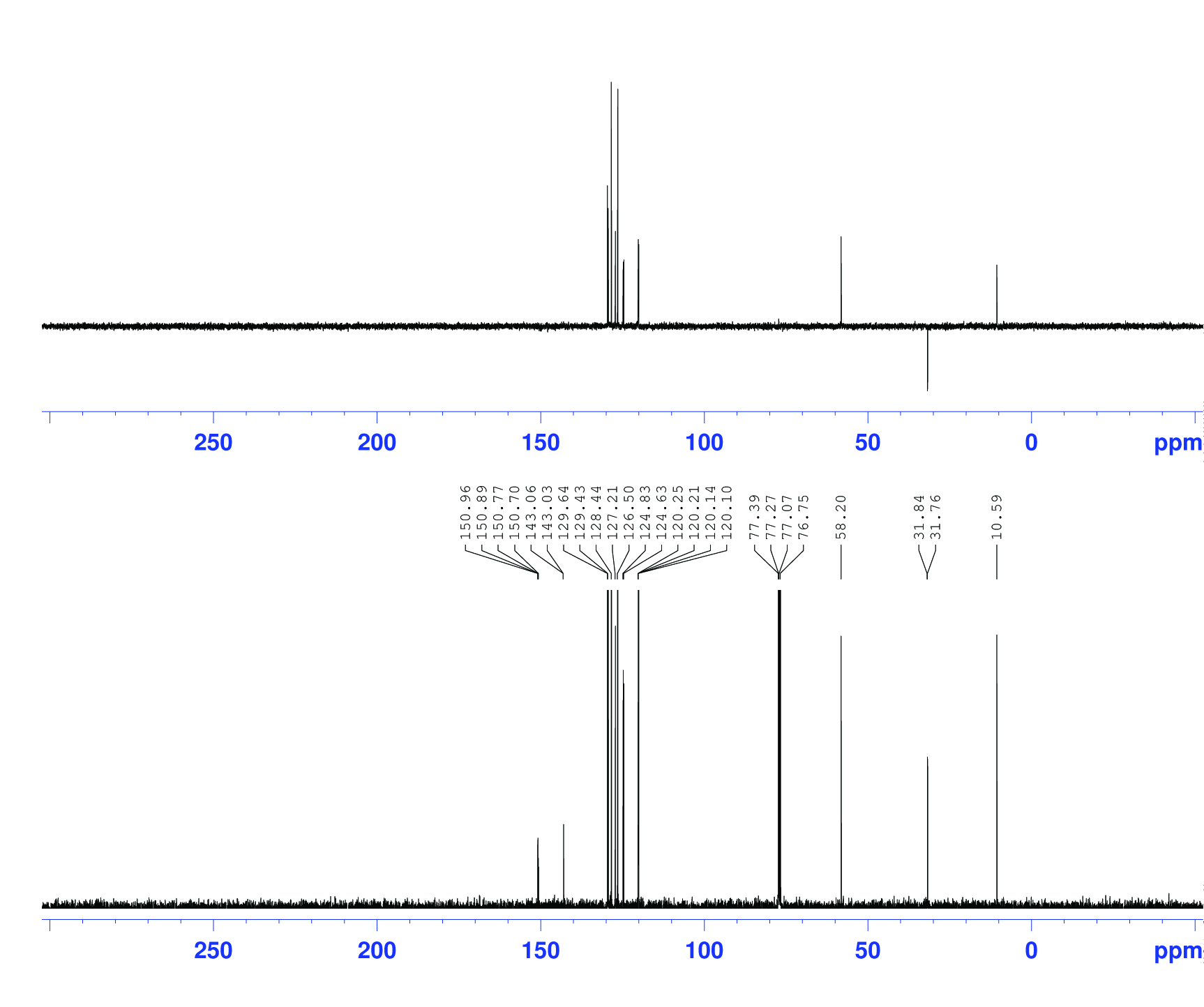


**Figure S5.** DEPT (top) and ^13^C{^1^H} (bottom) NMR spectra of **I** (in CDCl_3_).


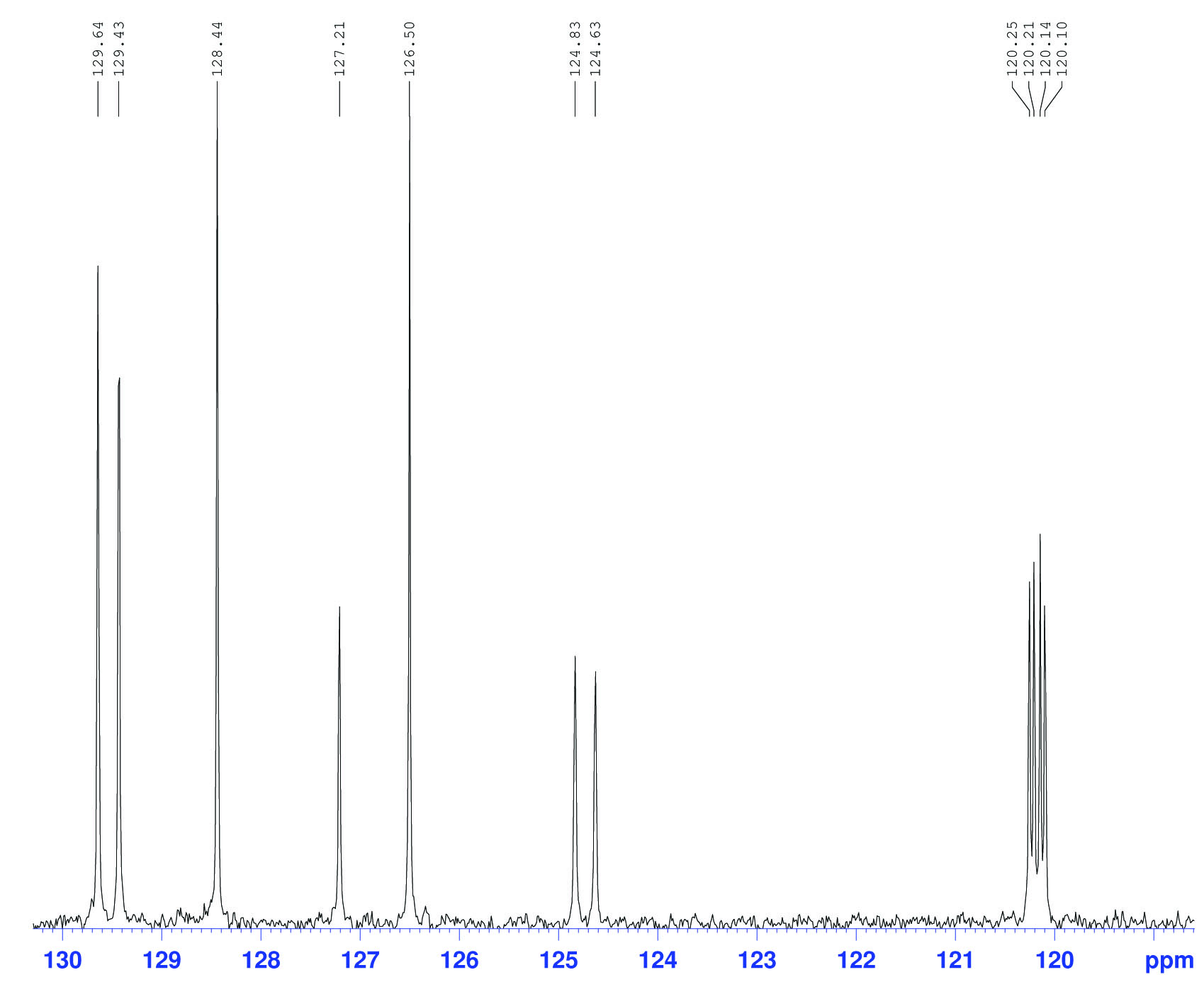


**Figure S6.** ^13^C{^1^H} NMR spectrum of **I** in the range of 120 to 130 ppm. The two doublets at about 120 ppm are related to the diastereotopic *ortho*- carbon atoms.


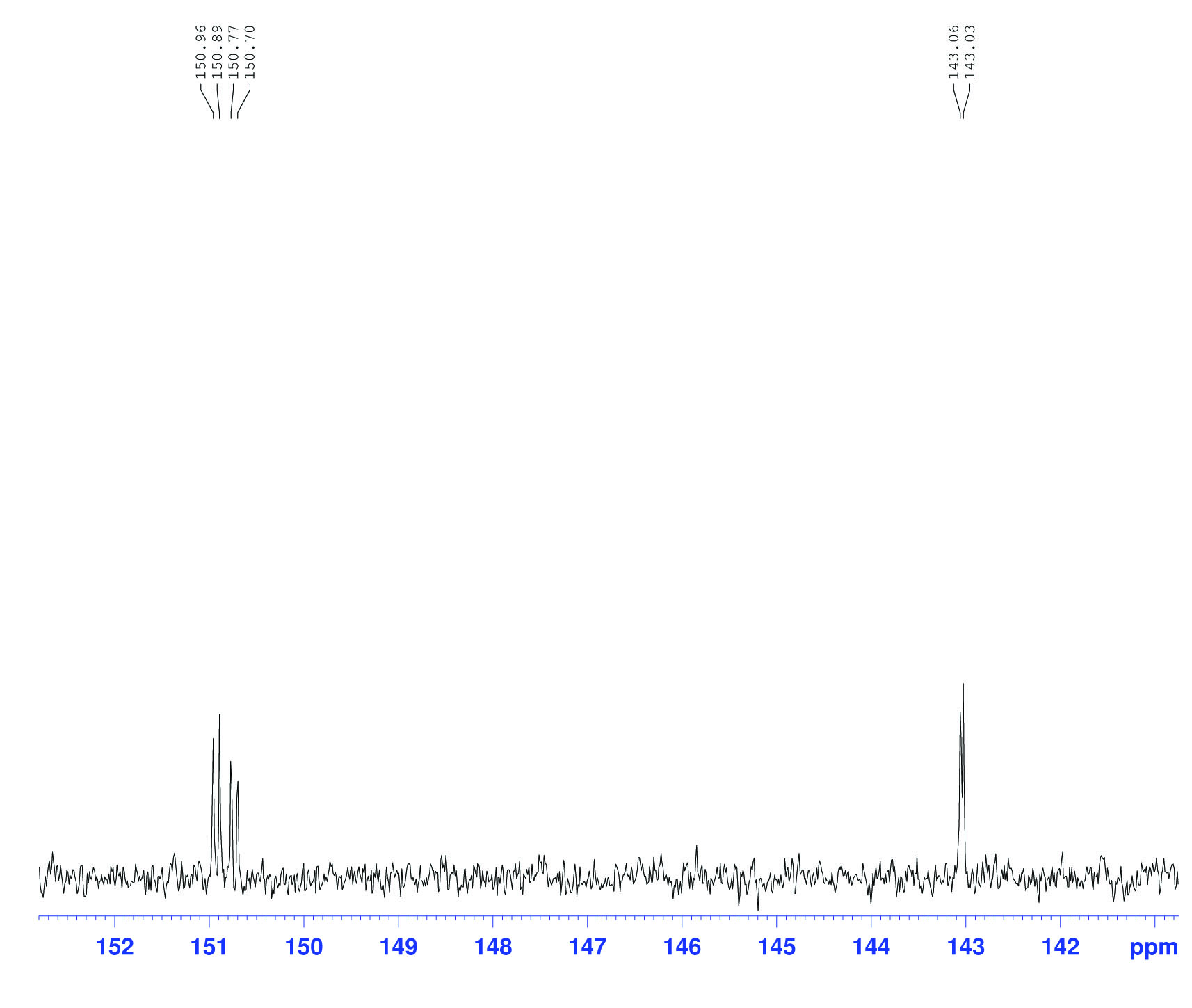


**Figure S7.** ^13^C{^1^H} NMR spectrum of **I** in the range of 142 to 152 ppm. The two doublets within 150 to 151 ppm are related to the diastereotopic *ipso* carbon atoms.


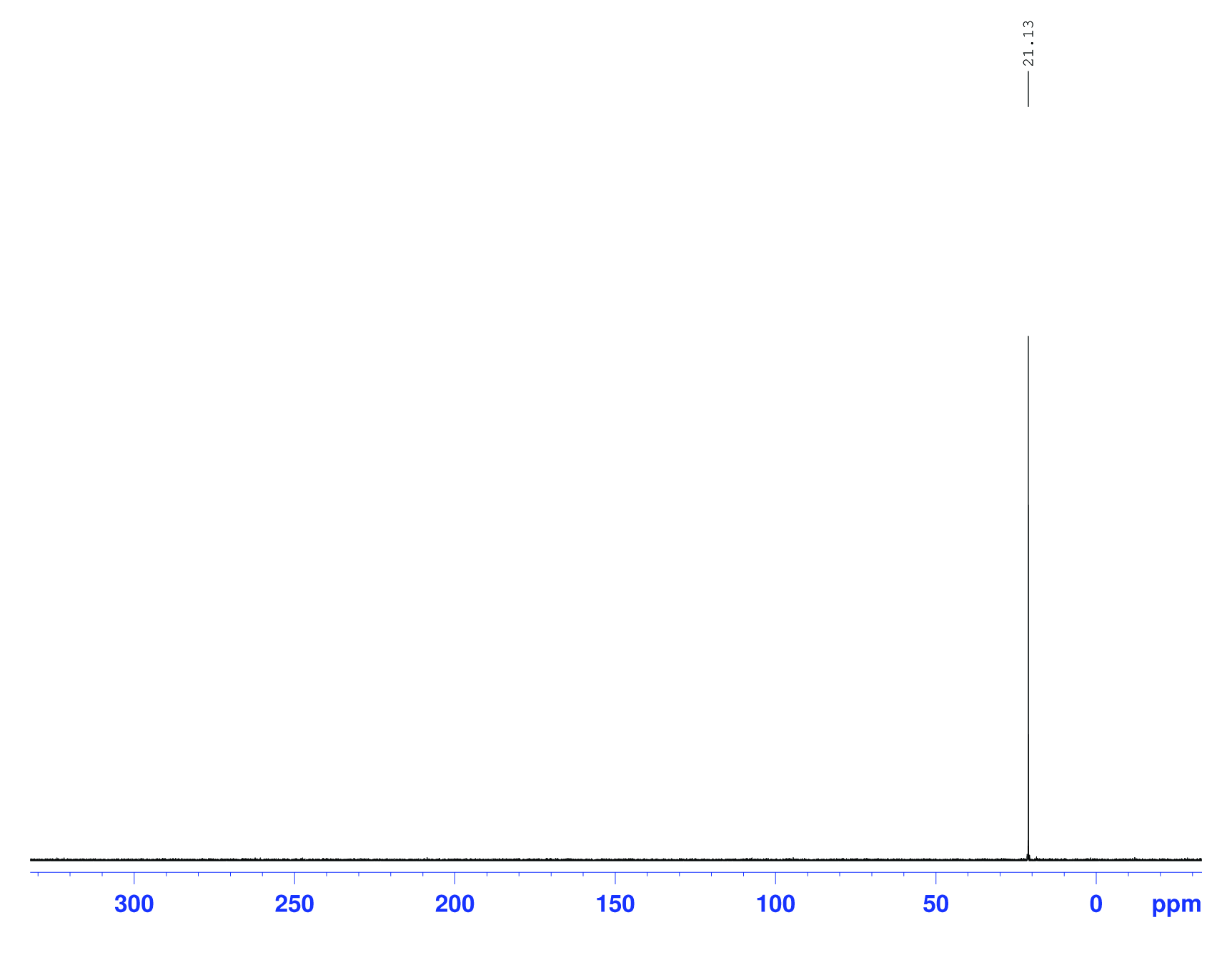


**Figure S8.** ^31^P{^1^H} NMR spectrum of **II** (in DMSO-*d*_6_).


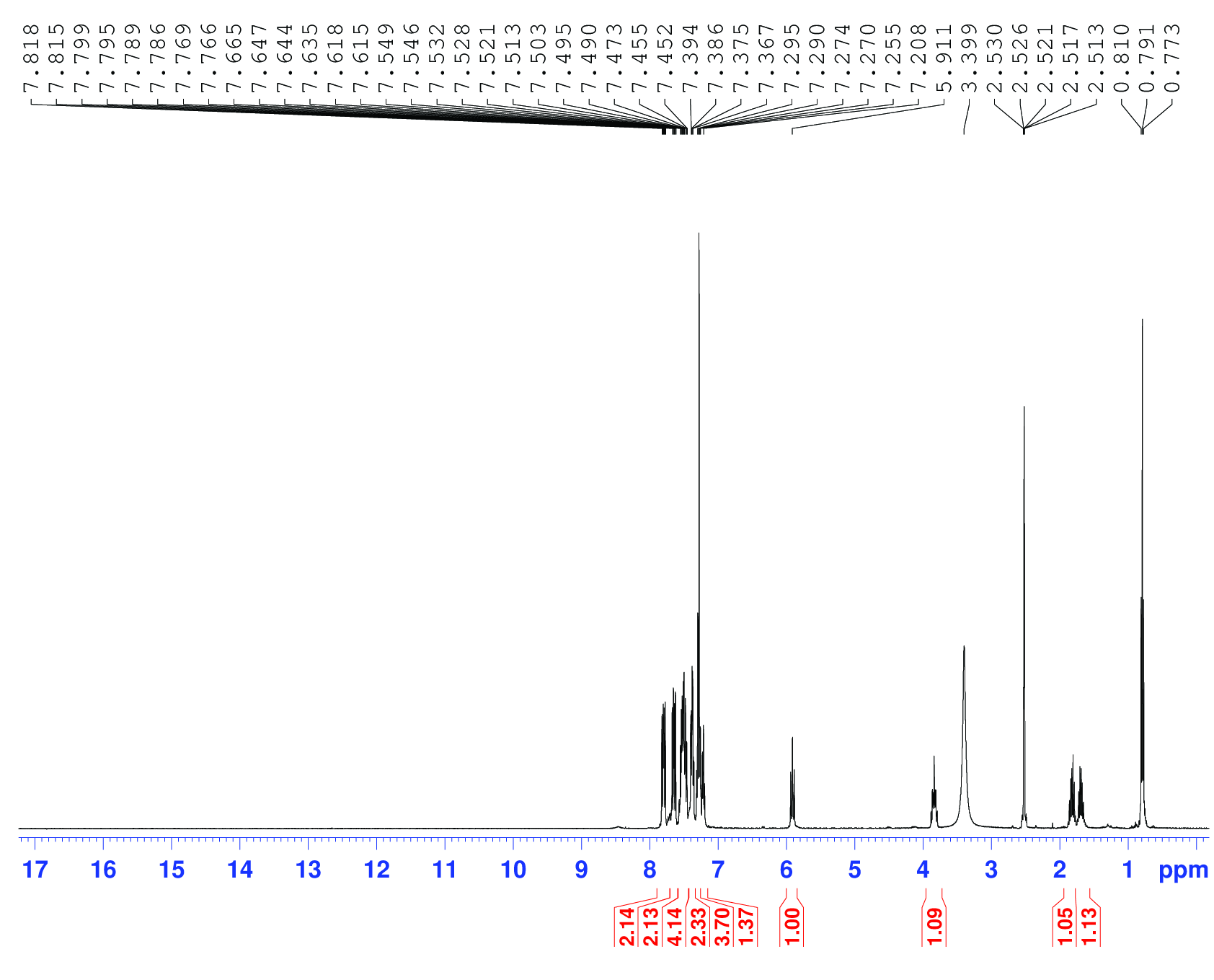


**Figure S9.** ^1^H NMR spectrum of **II** (in DMSO-*d*_6_).

**
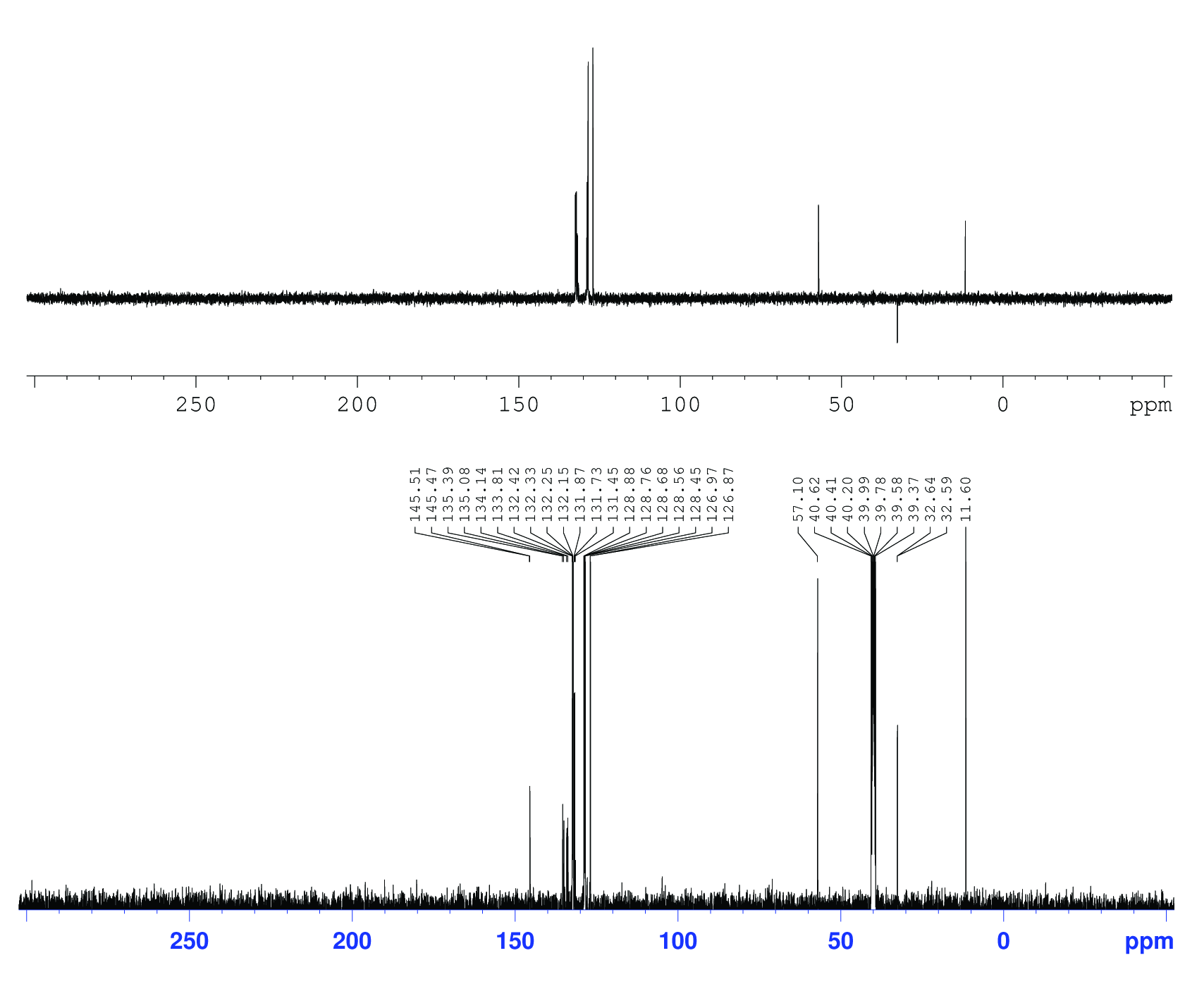
**

**Figure S10.** DEPT (top) and ^13^C{^1^H} (bottom) NMR spectra of **II** (in DMSO-*d*_6_).

**
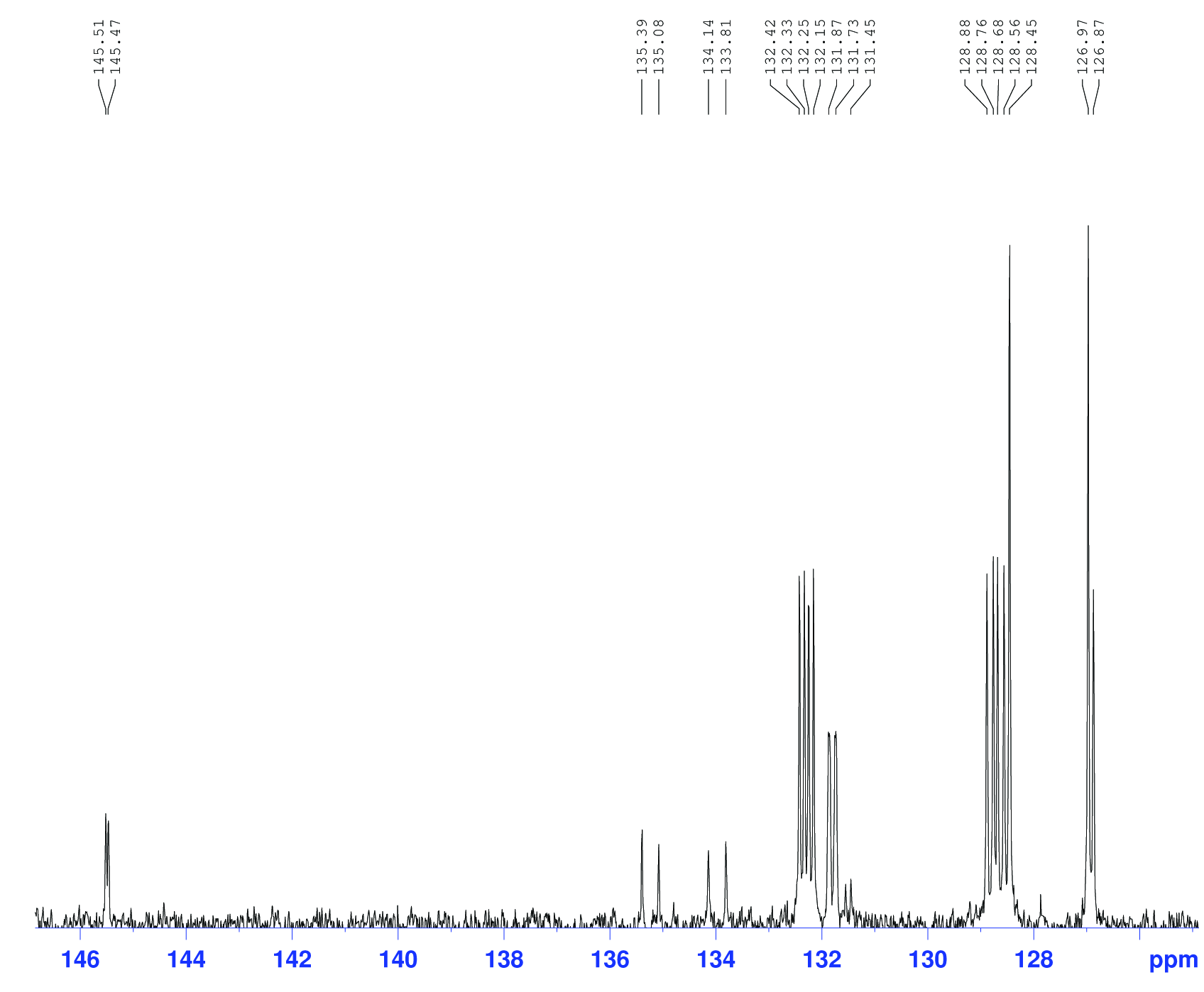
**

**Figure S11.** ^13^C{^1^H} NMR spectrum of **II** in the range of 127 to 146 ppm. The pairs of doublets at about 128 ppm (two doublets), below 132 ppm (two doublets), above 132 ppm (two doublets) and within 134 and 135 ppm (two doublets) are related to the diastereotopic *ortho*-, *para*-, *meta*- and *ipso*- carbon atoms, respectively. For ^1^*J* coupling, the branches at 133.81 and 135.08 ppm are considered for one doublet and the branches at 134.14 and 135.39 ppm for the other doublet. This assignment is based on the amount of previously calculated ^1^*J* coupling constant in achiral compounds, where only one doublet arisen from this coupling is observed.
